# Interleukin-1β and Cancer

**DOI:** 10.3390/cancers12071791

**Published:** 2020-07-04

**Authors:** Cédric Rébé, François Ghiringhelli

**Affiliations:** Platform of Transfer in Cancer Biology, Centre Georges François Leclerc, INSERM LNC UMR1231, University of Bourgogne Franche-Comté, F-21000 Dijon, France

**Keywords:** IL-1β, inflammasomes, immune cells, angiogenesis, metastasis

## Abstract

Within a tumor, IL-1β is produced and secreted by various cell types, such as immune cells, fibroblasts, or cancer cells. The IL1B gene is induced after “priming” of the cells and a second signal is required to allow IL-1β maturation by inflammasome-activated caspase-1. IL-1β is then released and leads to transcription of target genes through its ligation with IL-1R1 on target cells. IL-1β expression and maturation are guided by gene polymorphisms and by the cellular context. In cancer, IL-1β has pleiotropic effects on immune cells, angiogenesis, cancer cell proliferation, migration, and metastasis. Moreover, anti-cancer treatments are able to promote IL-1β production by cancer or immune cells, with opposite effects on cancer progression. This raises the question of whether or not to use IL-1β inhibitors in cancer treatment.

## 1. Introduction

The IL-1 family includes four main members, namely, IL-1α, IL-1β, IL-33, and IL-1 receptor antagonist (IL-1RA). IL-1α, IL-1β, and IL-33 are cytokine activators, while IL-1RA is an inhibitory cytokine [1]. IL-1 cytokines bind the type 1 receptor (IL-1R1), except IL-33, which binds IL-1R4 (ST2). While IL-1α or IL-1β fixation on IL-1R1 induces a downstream signaling cascade and the transcription of several genes involved in inflammatory and immune pathways, IL-RA fixation does not have the same effect [2].

IL-1α and IL-1β are encoded by two different genes, with a low degree of sequence homology. They are both synthesized as preform proteins, pro-IL-1α and pro-IL-1β. Pro-IL-1β needs to be converted into IL-1β (by inflammatory caspase cleavage) to be active, whereas pro-IL-1α is active, and its cleavage into IL-1α (by calpain) will modulate its activity [3]. IL-1α can be localized in the nucleus, where it acts as a transcription factor to regulate cell differentiation in normal cells, as well as neoplasia in cancer cells. However, when cells undergo cell death, such as necrosis, IL-1α translocates into the cytosol and is released into the extracellular space to act as an « alarmin » [3,4]. In contrast, the synthesis and processing of IL-1β are tightly controlled and require two signals, namely “priming” to allow transcription of the IL1B gene and the activation signal, which leads to activation of inflammasome complexes and inflammatory caspases to cleave pro-IL-1β into mature IL-1β [5].

Although IL-1β and IL-1α share similar transducing pathways, they have different expression and activation processes, explaining why they have different biological and physiological effects in many diseases, notably cancer.

### 1.1. IL-1β Production

**Figure 1:**
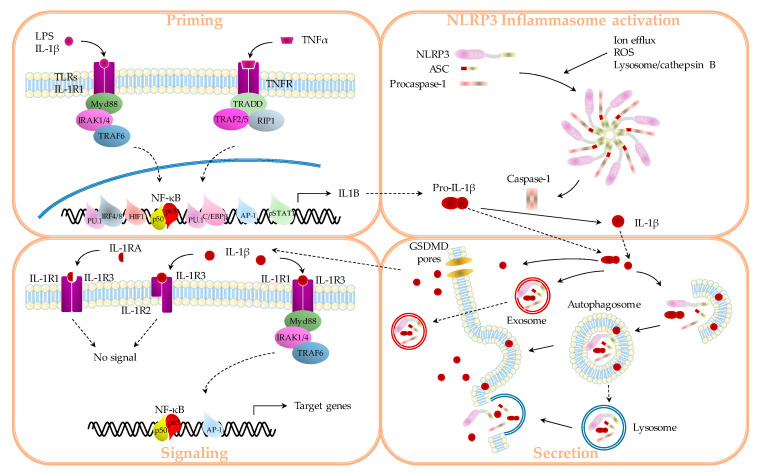
Different steps of interleukin (IL)-1β production and signaling: priming, NOD-LRR and pyrin containing protein 3 (NLRP3) inflammasome activation, secretion, and signaling. LPS, lipopolysaccharides; TLR, toll-like-receptor; TNF, tumor necrosis factor; TNFR, TNF receptor; TRADD, TNFR1-associated death domain; RIP, receptor interacting protein; IRAK, interleukin-1 receptor-associated kinase; Myd, myeloid differentiation primary response; TRAF, TNFR-associated factor; ASC, apoptosis associated speck-like protein containing a CARD domain; GSDMD, gasdermin D; ROS, reactive oxygen species; NF-κB, nuclear factor kappa-light-chain-enhancer of activated B-cells.

Within the tumor, IL-1β is produced and secreted by various cell types, such as immune cells, fibroblasts, or cancer cells. However, the mechanisms of IL-1β production have been most widely studied in immune cells, particularly in myeloid cells, such as macrophages. As mentioned above, IL-1β production requires two signals, namely “priming” and cleavage (Figure 1).

#### 1.1.1. “Priming”

“Priming” corresponds to the transcription of the Il1b/IL1B gene, and is induced mainly by activation of the toll-like-receptors (TLRs), namely lipopolysaccharides (LPS), but also by tumor necrosis factor (TNF) α, through the TNF receptor or IL-1β itself (Figure 1). TLRs and IL-1R1 recruit the same adaptor protein, myeloid differentiation primary response 88 (Myd88), through their intra-cellular domain, which in turn activates interleukin-1 receptor-associated kinase 1/4 (IRAK1/4) and the TNFR-associated factor 6 (TRAF6) pathway. TNFR recruits TNFR1-associated death domain (TRADD), which activates the TRAF2/5 and receptor interacting protein 1 (RIP1) pathway. All these signaling cascades are able to activate nuclear factor kappa-light-chain-enhancer of activated B-cells (NF-κB) [6,7]. As hypoxia is an important event within the tumor, hypoxia-induced factor 1 (HIF1) was shown to regulate Il1b/IL1B transcription. Other classical transcription factors were also shown to induce Il1b/IL1B expression in myeloid cells, such as CCAAT/enhancer binding protein (C/EBP-)β, Interferon response factors 4 or 8 (IRF4/8) and PU.1 or protein kinase C (PKC)/activator protein-1 (AP-1), directly or through Myd88 [6]. The STAT1 pathway was also shown to be required in macrophages to enable IL-1β production [8]. Finally, single nucleotide polymorphisms (SNPs) in the Il1b/IL1B gene or promoter can affect Il1b/IL1B transcription by inhibiting the fixation of the transcription factors described above or by allowing the fixation of repressor factors.

After transcription/translation, pro-IL-1β is produced as an inactive, 31kDa protein that needs to be cleaved into 17kDa IL-1β to become active.

#### 1.1.2. Inflammasomes

To be activated, pro-IL-1β needs to be cleaved by proteases. Although cleavage by cathepsin G or elastase in neutrophils has been described, yielding low-activity IL-1β, caspases are the main proteases that can provide fully-active IL-1β. Caspase-8 has been shown to cleave IL-1β in vitro and in specific conditions. However, the most important enzyme involved in IL-1β maturation remains caspase-1, which has been reported to be activated via numerous sources [9].

Caspase-1 activation occurs via recruitment to multi-protein complexes called inflammasomes. These intracellular complexes are all composed of a receptor and an adaptor, allowing recruitment and activation of pro-inflammatory caspases [5]. 

The receptors, called nucleotide-binding oligomerization domain-containing protein (Nod)-like receptors (or NLR), recognize a wide variety of stimuli referred to as pathogen-associated molecular patterns (PAMPs) (Figure 1). The NLR family is characterized by the presence of several specific domains. All of these proteins have a central NACHT ((NAIP (neuronal apoptosis inhibitor protein), C2TA (MHC (major histocompatibility complex) class 2 transcription activator), HET-E (incompatibility locus protein from Podospora anserina), and TP1 (telomerase-associated protein)) domain, responsible for complex activation, via ATP-dependent oligomerization. At the C-terminal, the leucine-rich domain (LRR) is involved in ligand detection and in complex self-regulation. The N-terminal is either a pyrin domain (PYD) or a caspase recruitment domain (CARD) involved in protein–protein interactions for signal transduction. Thus, activated receptors can recruit either pro-caspases (via the CARD) or an adaptor protein (via the PYD), which in turn will recruit a pro-caspase. The NLR receptors are divided into four families, with many members, according to the N-terminal domain composition. However, the NOD-LRR and pyrin containing protein 3 (NLRP3) is the most important receptor, and is responsible for caspase-1 activation under sterile conditions [5,10].

Prior to its activation, the expression of NLRP3 is under the control of NF-κB activation, like IL-1β. NLRP3 is activated by a wide variety of stimuli and by three non-exclusive pathways, with possible crosstalk. The first is intracellular K^+^ efflux induced by binding of extra-cellular ATP to its receptor P2X7. The second involves phagocytosis of crystalline structures and subsequent lysosome damage. Lysosomal content, especially cathepsin B, will then activate NLRP3 through direct interaction. The third pathway involves an increase in reactive oxygen species (ROS) synthesis. All these steps will converge to NLRP3 activation, recruitment of ASC (apoptosis associated speck-like protein containing a CARD domain) and pro-caspase-1, and IL-1β and IL-18 maturation [11,12].

#### 1.1.3. Secretion

The mechanisms that lead to IL-1β secretion are not clear (Figure 1). This might be explained by the existence of several pathways that may or may not co-exist, depending on the context (Figure 1). Studies of IL-1β secretion used different experimental settings, such as in vivo, ex vivo, or in vitro cultures; different cellular models (primary cells, cell lines) and cell types (macrophages, fibroblasts, neutrophils); several activators that induce cell death or not (pyroptosis, necrosis); as well as different techniques to detect IL-1β (ELISA, Western blot, IL-1 HEK-blue cells). Moreover, techniques used to investigate IL-1β secretion pathways, such as electronic microscopy, immunofluorescence, or the use of more or less specific inhibitors of intra-cellular traffic, only give clues and not a real explanation. Actually, analyses are made on single cells at a specific time point and may make impossible to determine whether one or two mechanisms co-exist. In general, IL-1β is released through vesicles (autophagolysosomes, microvesicles, or exosomes) or through membrane permeability (gasdermin D (GSDMD) pores, membrane rupture).

Pro-IL-1β and IL-1β are localized in the cytoplasm and a fraction is sequestered in vesicles identified as endosomes/lysosomes, via an unidentified mechanism. A part of lysosomal IL-1β is targeted for degradation, while a fraction is saved for further exocytosis and secretion, through fusion with the plasma membrane [13,14]. However, IL-1β may be protected from the acidic pH of lysosome, to avoid its degradation. This rescue is enabled thanks to autophagy, a process that encapsules damaged organelles or proteins in a double membrane structure called autophagosome. Autophagosomes generally fuse with lysosomes to proteotically degrade their content. In this context, it has been reported that IL-1β can localize between the two layers of autophagosomes, possibly explaining why IL-1β is not degraded [15]. Autophagosomes can also fuse with IL-1β-containing endosomes to undergo exocytosis and IL-1β release out of the cell [13,14]. Multi vesicular bodies containing IL-1β (but also caspase-1 and inflammasome components) can fuse with lysosomes for degradation of their content or with plasma membrane to form and release exosomes [16].

Once cleaved, IL-1β exhibits an overall positive charge to enable it to colocalize with negatively charged phosphatidylinositol 4,5-bisphosphate (PIP2) in the plasma membrane. Then, shedding of microvesicles from plasma membranes makes it possible for IL-1β to be released out of the cell [17].

As well as cleaving pro-IL-1β, activated caspase-1 can also trigger a type of inflammatory cell death called pyroptosis. This type of cell death is characterized by inflammatory caspase activation (caspase-1 and/or caspase-11/4/5) and LDH (lactate dehydrogenase) release through membrane permeabilization. This latter event was recently shown to be mediated by gasdermin D (GSDMD) cleavage. Once cleaved by caspase-1, -11, or -5, the N-terminal fragment of GSDMD oligomerizes into ring-shaped structures to form membrane pores. These pores enable the exit of mature IL-1β (the proform being too big). They also allow the entry of sodium and water. If the activation signal and the number of GSDMD pores are low, membrane fusion can patch the pores. On the contrary, if the signal and the number of GSDMD pores are high, the sodium and water entry will induce membrane rupture, allowing the release of the cytosolic content such as pro-IL-1β and IL-1β. However, pro-IL-1β release is not without consequence if extra-cellular proteases cleave it into mature form [18,19].

The release of IL-1β and its preform pro-IL-1β into vesicles raises questions about its stability and detection. Actually, IL-1β has a very short half-life [20] and its packaging into vesicles can allow it to resist degradation and act at sites distant from the production site. As IL-1β can be encapsulated into vesicles, and pro-IL-1β can be found in the supernatant, the detection of active IL-1β by classical methods such as ELISA can be compromised [21,22,23]. To confirm the presence of mature IL-1β, complementary methods, such Western blot on cell supernatants or HEK-blue cells, to detect active IL-1β should be used. However, the incapacity to detect IL-1β would not assure its absence.

### 1.2. IL-1β Signaling

IL-1β binds to IL-1R, which belongs to the superfamily of TLR/IL-1R, characterized by the presence of an intra-cellular TIR (TLR/IL-1R) domain (Figure 1). More particularly, IL-1β can bind to IL-1R1 and IL-1R2, which present three extra-cellular Ig binding domains and are associated with the highly homologous IL-1R accessory protein (IL-1RAcP or IL-1R3). However, the transmission of a signal after IL-1β binding to its receptors is not a simple matter. In fact, IL-1R2 has no TIR domain and acts as a decoy receptor. Moreover, IL-1RA can also bind IL-1R1 without inducing an activator signal, taking the place of IL-1β [6,24]. Thus, only IL-1β/IL-1R1/IL-1RAcp will enable transmission of a signal. This raises the question not only of the amount of IL-1β produced, but also of the level of IL-1Rs expression on target cells and the level of IL-1RA.

Once activated, IL-1R/IL1-RAcP can recruit Myd88 through TIR domains present in the intra-cellular domain of IL-1R and on the C-terminal domain of Myd88. Then, MyD88 associates with IRAK 4, IRAK1, and/or IRAK2. IRAK4 in turn phosphorylates IRAK1 and IRAK2 to enable their association with TRAF6. TRAF6 serves as a platform to recruit and activate the transforming growth factor β-activated kinase 1 (TAK1). TAK1 will activate either p38 and JNK (c-Jun N-terminal kinase), leading to activation of transcription factor AP-1, or the IKK (inhibitor of NF-*κ*B kinase) complex, composed of IKKα, IKKβ, and IKKγ. The IKK complex catalyzes phosphorylation and subsequent degradation of IκB, rendering NF-κB (i.e., p50/p65) free to translocate from the cytosol to the nucleus and to activate NF-κB-dependent genes [25].

The activation of p38/JNK and NF-κB leads to the transcription of target genes involved in several biological processes, depending on the cell type stimulated by IL-1β.

## 2. IL-1β as a Cancer Marker?

The importance of IL-1β can first be considered by its impact on cancer development or progression. This evaluation can be performed by quantifying IL1B mRNA or IL-1β protein expression or by measuring gene polymorphisms that may influence its expression (Table 1).

Using the cancer genome atlas (TCGA) database, breast cancer patients with high levels of mRNA expression of IL1B were shown to have a better prognosis than those with low levels [30]. Similarly, the quantification of IL1B mRNA by qPCR in cervical cancer biopsies showed an increase in the risk of progression of pre-neoplasic lesions in women with lower IL1B expression [31]. On the contrary, TCGA analysis on glioma patient samples shows that high expression of “IL1B-signature” is correlated with high expression of CD133 (a marker of glioma aggressiveness) and associated with poor prognosis [32]. These discrepancies might be explained by the fact that mRNA expression was studied at a single time point and that these analyses did not consider the patient’s cancer stage, or whether they had received treatment. Moreover, IL1B mRNA expression cannot predict its maturation by inflammasomes.

In this concept, immunohistochemical analyses showed that upregulation of ASC, caspase-1, IL-1β, AIM2, RIG-I, and NLRP3 expression correlated with better local recurrence-free survival and disease-free survival of nasopharyngeal carcinoma patients [26]. Again, this increased expression does not reflect IL-1β activity.

Finally, IL-1β levels can be measured in patient plasma or serum by ELISA. IL-1β is significantly overexpressed both at mRNA and protein levels in gastro-esophageal cancer or squamous cell carcinoma samples compared with mucosa from controls [56,57,58]. High IL-1β levels are associated with shorter overall and progression-free survival for non-small cell lung cancer (NSCLC) patients treated with platinum-based combination chemotherapy or with chemotherapy/bortezomib and for pancreatic cancer patients treated with gemcitabine [27,28,29].

Polymorphisms on the IL1B gene can be associated with variation in IL-1β expression. For example, *IL1B-511 C > T* (rs16944) and *IL-1β-31 C > T* (rs1143627) T alleles are associated with an increase in IL-1β serum concentration in cervical and gastric cancer patients [59,60,61], or in the supernatant of cells harboring rs1143627 [62]. Conflicting results were obtained concerning *IL1B-511* (rs16944) homozygote C/C genotypes, suggesting a low expression of IL1B. In one study, it was associated with the risk of ovarian cancer, while in two others, it was not [33,34,35]. Similarly, C/C genotypes may or may not be associated with a higher risk of lung cancer, depending on the studies [36,37]. *IL1B-511T* carriers, suggesting higher expression of IL1B, present a higher risk of developing gastric cancer [38,39,40,41,42,43,44,45], or not [46]. *IL1B-511T* carriers present a higher risk of developing cervical cancer, acute myeloid leukemia, or chronic myeloid leukemia [47,48,49,50]. Concerning breast cancer, no association was shown between *IL1B-511* (rs16944) and the risk of breast cancer development [63]. *IL-1β-31* (rs1143627) T allele is associated with an increased IL1B expression. The T/T genotype was associated with a higher risk of breast cancer [51,52], lung cancer [36,37], cervical cancer [48], hepatocellular carcinoma [53], or osteosarcoma [54] in various studies. On the contrary, C allele carriers have a higher risk of developing gastric cancer [45]. For *IL-1β-1464 G > C* (rs1143623), the G allele has decreased binding ability, suggesting weaker promoter activity [64]. It is associated with renal cell carcinoma [55].

With all these results, it is difficult to certify whether IL-1β expression or IL1B polymorphisms can predict the outcome of cancer patients.

## 3. Pro-and Anti-Tumor Effects of IL-1β

IL-1β has been shown to play a role in many physiological events. It can modulate gene expression and cytokine production, regulating cellular adhesion and migration, angiogenesis, or immune response. However, the repercussions on the course of cancer are complex, and both positive and negative functions of IL-1β have been described. These observed discrepancies make IL-1β a possible target that may need to be taken in consideration, depending on the cancer type and the anti-tumor treatments.

### 3.1. IL-1β Effects on Cancer Occurrence

IL-1β has been shown to be upregulated in many solid tumors, including melanoma, colon, lung, breast, or head and neck cancers and is associated with poorer prognosis. While its role in carcinogenesis is well described for some cancer types, its implication in other types is not as well elucidated.

#### 3.1.1. Skin Cancers

NLRP1 gain-of-function mutations are responsible for constitutive secretion of IL-1β by keratinocytes, which enable skin inflammation and epidermal hyperplasia, and a predisposition to skin cancer [65]. Human metastatic melanoma samples and human cell lines were described to constituvely express and secrete IL-1β [66]. Using B16 melanoma or 3-methylcholanthrene (3-MCA)-induced skin cancer models, it has been shown that the incidence of tumor development in mice was impaired in IL-1β-deficient or in IL-RA-treated animals [67,68]. On the contrary, the incidence of mice-bearing tumors was improved in IL-1RA-deficient animals [68]. In contrast, another study showed in the B16-F10 model that blocking IL-1β with an antibody increased tumor appearance in wild type (WT) mice, thus suggesting that IL-1β was protective in this context [69].

#### 3.1.2. Colon Cancer

High amounts of IL-1β and IL-1α were detected in a murine adenomatous polyposis coli (APC) colon cancer model [70]. Contrasting effects of IL-1β have been described on colon cancer incidence. This may partly be because of the use of mice deficient in NLRP3 inflammasome components, which are responsible not only for IL-1β production, but also for IL-18 production [71,72,73]. Another possible explanation is that IL-1β targets several cell types. Using disruption of IL1-R1 on different cell types in a mouse model, it was recently shown that IL-1R1 deficiency in epithelial cells reduces tumorigenesis in an APC model, while IL1-R1 deficiency in neutrophils increases bacterial invasion and tumor aggressiveness [70]. This study proposed dichotomous effects of IL-1, without differentiating between IL-1β and IL-1α.

In vitro, IL-1β was shown to upregulate miR-181a expression in human colon cancer cells, through NF-κB, which is responsible for phosphatase and tensin homolog (PTEN) repression and cell proliferation induction [74]. A similar effect of IL-1β on colon cancer cell proliferation was shown via inactivation of glycogen synthase kinase (GSK)3β, leading to activation of the Wnt pathway and tumor growth [8].

#### 3.1.3. Lung Cancer

The level of IL-1β in bronchoalveolar lavage is higher in patients with lung cancer than in patients with benign lung disease [75]. IL-1β was shown to promote carcinoma by repressing miR-101 expression through a cyclooxygenase 2 (COX2)/HIF1α pathway. MiR-101 inhibits malignant transformation and cancer progression by negatively regulating oncogene expression. Thus, IL-1β/miR-101 is a new regulatory axis of pathogenic inflammatory signaling in NSCLC [76].

#### 3.1.4. Breast Cancer

It has long been established that there is IL-1β protein expression within human breast tumor samples [77]. Moreover, IL-1β is upregulated in breast neoplasm initiation and development [78], while IL-1R and IL-1β variations have also been related to breast tumorigenesis [79]. One possible explanation is that IL-1β increases IL-6 production through a transglutaminase 2/NF-κB pathway. This leads to an increase in luminal-type breast cancer cell aggressiveness. This can be inhibited using an anti- IL-1β or an anti-IL-6 [80]. Another pathway has been described, using the fibroblast growth factor receptor 1 (FGFR1)-induced murine mammary carcinoma model. It implicates IL-1β-mediated expression of COX-2, which is responsible for early-stage mammary lesions [81]. The potential utility of inhibiting IL-1β was underlined by studies using deficient mice or anti-IL-1 antibody, suggesting that IL-1β in the tumor environment contributes to breast tumor progression [82,83].

#### 3.1.5. Gastric Cancer

The use of a human IL-1β fused to a signal peptide to specifically induce its expression in mouse stomach epithelial cells led to the development of spontaneous gastric inflammation; pre-neoplastic lesions; and, in some cases, tumors, suggesting an initiator role of IL-1β [84]. Moreover, in gastric cells infected with *Helicobacter pylori,* yes-associated protein 1 (YAP1) displays nuclear translocation and works with TEAD to activate transcription of IL1B. The IL-1β thus produced displays YAP1-mediated cell proliferation [85]. To transduce this proliferating signal, IL-1β may bind to its receptor and activate NF-κB, which initiates JNK signaling, causing gastric cancer development [74,86,87].

#### 3.1.6. Oral Cancers

Salivary IL-1β was described to be significantly higher in oral cancer patients than in a control group [88]. In oral squamous cell carcinoma, IL1B is overexpressed in tumors as compared with non-tumor matched samples. In mice, induction of oral malignancy by 4-Nitroquinolin-1-oxide (4-NQO) and arecoline triggers pro-IL-1β expression, which is proportional to cancer severity [89].

#### 3.1.7. Pancreatic Cancer

In human pancreatic ductal adenocarcinoma (PDAC) samples, high stromal IL-1β expression is associated with poor overall survival of patients [90]. In mice, IL-1β involvement in cancer incidence was addressed, using IL-1β expression specifically in the pancreas, via the elastase promoter. While the expression of IL-1β resulted in chronic pancreatitis, mice only developed acinal-ductal metaplasia [91]. Perhaps the use of a carcinogen inductor, such as dimethylbenzanthracene, would make it possible to prove the importance of IL-1β in the appearance of pancreatic carcinoma.

Further studies are warranted to elucidate these observed discrepancies. Differences might be because of the means used to invalidate IL-1β, that is, KO mice with no IL-1β in the host, but with tumor cells producing IL-1β, or neutralizing antibody with a decrease, but not inhibition of both host and tumor IL-1β.

#### 3.1.8. Ovarian Cancer

Urinary and serum levels of IL-1β tend to be more elevated in patients with epithelial ovarian cancer than in healthy women [92]. In the 2780 ovarian cancer cell line, IL-1β induces the expression of matrix metalloproteinase (MMP)8, a factor implicated in cancer progression [93].

#### 3.1.9. Prostate Cancer

High-score values for IL-1β or low-score values for interferon (IFN)β (both measured by immunohistochemistry (IHC)) were significantly associated with biochemical recurrence of prostate cancer [94]. Moreover, IL-1β and IL-1R2 (the decoy receptor) high expression and IL-1R1 low expression are associated with higher progression free survival (PFS) [95].

In vitro monocytic-derived IL-1β inhibits LNCaP prostate cancer proliferation or induces apoptosis [96,97,98]. Moreover, IL-1β resistant LNCaP cells (obtained after long exposure to IL-1β) become resistant to many chemotherapeutic drugs and have a more important capacity to develop tumors in mice [98]. IL-1β has an antiproliferative effect on prostate cancer cells, enhanced by coculture with normal fibroblasts, through IL-6 [99]. Mechanistically, IL-1β can induce prostate tumor progression by several pathways. Through NF-κB, IL-1β induces the activation of epithelium-specific ETS (E26 transformation-specific) ESE1 (or E74-like factor (ELF3)), two ETS family members responsible for prostate cancer malignancy and associated with a poor prognosis for patients [100]. IL-1β can also induce the expression of endothelin 1 (ET-1), which is implicated in prostate tumor progression [101]. Finally, IL-1β induces the expression of matrisylin in human LNCaP prostate cancer cells, a metalloprotease involved in cancer progression [102].

Androgen inhibition belongs to the therapeutic arsenal to treat prostate androgen receptor (AR) positive cancers. It has been shown that IL-1β decreases AR expression, which may interfere with anti-androgen therapies’ efficiency [93,103,104]. An amplification loop may exist, as AR^-^ cancers cells express high levels of IL-1β, while AR^+^ cells do not and androgen-deprivation drugs, that is, leuprolide and bicalutamide, inhibit prostate cancer cells’ mediated IL-1β secretion by peripheral blood mononuclear cells (PBMC) in vitro [105,106].

#### 3.1.10. Mutational Status

In addition to cancer types, IL-1β can affect or can be affected by common cancer-associated mutations.

In acute lymphoblastic leukemia, KRAS (Kirsten rat sarcoma viral oncogene homolog) G12D mutation is responsible for the binding of cAMP response element binding (CREB) on IL1B promoter and increases the expression of IL-1β in these cells [107]. KRAS G12D expression in murine bone-marrow cells leads to NLRP3 inflammasome activation and IL-1β expression and to myeloproliferation. IL-1RA or NLRP3 inhibition reverses the effects of KRAS mutation on myeloproliferation [108]. Moreover, overexpression of IL-1β in KRAS G12D mutant mice accelerates the development of PDAC through autocrine activation of IL-1R1-mediated epithelial cell proliferation and an increased level of immunosuppressive PD-L1^+^ B-cells [109]. This finding is correlated with the fact that myeloid-derived IL-1β induces NF-κB activation more importantly in KRAS mutant (G12C or G13R) cancer cells than in WT cells, leading to drug resistance [110]. Thus, an amplification loop between IL-1β and mutated-KRAS seems to increase cancer progression and drug resistance.

Expression of mutated BRAF (v-raf murine sarcoma viral oncogene homolog B1) V600E mutation induces the transcription of IL1A and IL1B in papillary thyroid carcinoma cells, melanocytes, and melanoma cell lines and this induction can be inhibited by vemurafenib [111,112,113]. On the contrary, this BRAF(V600E) inhibitor increases DC-mediated IL-1β production [114]. However, the impact of BRAF(V600E) on IL-1β production and cancer evolution remains to be studied.

IL-1β treatment leads to a decreased PTEN expression, PI3K/AKT signaling activation, and to the induction of epithelial to mesenchymal transition (EMT) in NSCLC cells [115]. IL-1β induces the expression of miR-425, miR-181a, and miR-181b through NF-κB, in gastric cancer, colon cancer, and osteosarcoma cells, respectively. These miRNA repress PTEN expression, leading to apoptosis inhibition and proliferation-associated cancer cell growth [74,116,117]. PTEN expression in myeloid cells dictates NLRP3 inflammasome activation and IL-1β expression. Then, it allows mitoxanthrone anti-cancer activity in MC0205 fibrosarcoma model in mice. Moreover, in breast cancer patients, PTEN expression is correlated with IL-1β expression and to anthracyclines-based adjuvant chemotherapy sensitivity [118].

Ovarian cancer cells communicate with cancer-associated fibroblasts (CAFs) through IL-1β to downregulate p53 expression in these cells to generate a pro-tumorigenic inflammatory microenvironment [119]. Similarly, downregulation of IL-1β, IL1-R1, or Myd88 increases p21 and p53 in human melanoma cells [120]. On the contrary, the p53 status seems to regulate IL-1β response. WT p53 increases IL-1RA expression, which represses colon and breast cancer cells proliferation in vitro and tumor growth in vivo, while mutant p53 represses IL-1RA expression, allowing IL-1β effects [121]. Wnt secretion by p53-deficient breast cancer cells activates IL-1β production by macrophages. IL-1β then activates neutrophils to dampen CD8-mediated anti-tumor immune response [122].

P73 and, more particularly, Tap73, a constitutively active p73, is able to increase the transcription of IL1B and CASP1 in lung and breast cancer cells, allowing these cells to produce IL-1β [123]. The impact on cancer has to be defined.

BRCA1 (breast cancer 1) 185delAG mutation in ovarian epithelial cells allows IL-1β expression [124]. BRCA1 helps the sensing of herpes virus DNA and the activation of caspase-1 and IL-1β production [125]. However, the consequences of IL-1β in WT or mutated BRCA1 on cancer initiation or progression remain to be investigated.

### 3.2. IL-1β Effects on Tumor Immune Response

**Figure 2:**
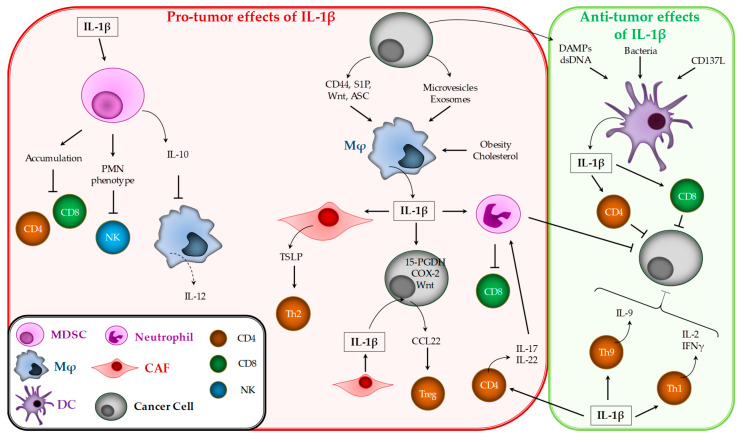
IL-1β effects on pro- and anti-tumor immune response. TSLP, thymic stromal lymphopoietin; CAF, cancer-associated fibroblast; PGDH, hydroxyprostaglandin dehydrogenase; MDSC, myeloid-derived suppressor cells; PMN, polymorphonuclear; COX, cyclooxygenase; DAMPs, danger-associated molecular patterns; CCL, C-C motif chemokine ligand; IFN, interferon; NK, natural killer.

As seen below, the fact that IL-1β can be produced endogenously and/or by cancer cells highlights the importance of the microenvironment, and more particularly immune cells, in IL-1β-mediated effects (Figure 2).

#### 3.2.1. Myeloid-Derived Suppressor Cells (MDSCs)

MDSCs form a population of immature myeloid cells with the ability to dampen T-cell activation [126]. These cells have been shown to markedly expand in lymphoid organs and blood in tumor-bearing mice [84]. In addition, the frequency of MDSCs is increased in the blood of patients with different types of cancers [127,128]. In mice and humans, MDSCs are one of the major suppressors of antitumor immunity, mainly by inducing antigen-specific MHC class I restricted tolerance of CD8^+^ T-cells [129].

The importance of IL-1 in MDSC accumulation came from a study showing that tumor bearing IL-1R1-deficient mice presented decreased tumor growth and fewer MDSCs [130]. However, this work did not discriminate the effects of IL-1α and IL-1β.

Overexpression of IL-1β in gastric cancer or fibrosarcoma models leads to accumulation of MDSCs at the tumor site. The inhibition of IL-1, using IL-1RA, decreases or suppresses MDSC accumulation at the tumor site and inhibits tumor development in these models [84,131].

Overexpression of IL-1β in mammary 4T1 tumor cells can modify MDSC phenotype (more CD8, CD80, CD83, and CD14 expression and lower CD44 and B220) in vivo, while it does not change their capacity to dampen CD4 and CD8 T-cells’ activation/proliferation [132]. This suggests that the effect of IL-1β relies more on the accumulation of MDSCs than on increased immunosuppressive activity. An effect of IL-1β on the different subtypes of MDSCs has been observed. In the mammary 4T1 tumor model, overexpression of IL-1β (in the tumor or the host) or the invalidation of IL-1RA led to an accumulation of Ly6C negative MDSCs, that is, polymorphonuclear (PMN)-MDSCs, whereas blocking IL-1β decreases the number of MDSCs. The consequences of high IL-1β expression are a decrease in functional natural killer (NK) cells and increased tumor growth [133]. However, the direct effect of IL-1β on MDSCs was not studied in this work. It was shown that MDSCs from mammary 4T1 tumors do not express IL-1R1 [132], suggesting that these cells cannot respond directly to IL-1β.

IL-1β-induced inflammation increases IL-10 production by MDSCs and activates MDSCs, which are more effective at down-regulating macrophage production of IL-12 as compared with MDSCs isolated from less-inflammatory tumor microenvironments [134].

#### 3.2.2. Macrophages

Tumor-associated macrophages (TAMs) compose a heterogeneous family that may be classically divided into M1 and M2 macrophages. This classification is based on the capacity of M1 to produce nitric oxide synthase/IL-12/TNF-α and to promote Th1 responses, while M2 produce arginase-1/IL-10/TGF-β to support Th2-associated effector functions [135,136]. However, a spectrum of polarization exists in the tumor with macrophages sharing markers or expressing atypical markers. Macrophages, and more particularly those of the M1 type, are the cells most commonly described to be able to produce IL-1β under several stimuli, as their differentiation in vitro is induced by macrophage colony-stimulating factor (M-CSF), LPS, and IFNγ [137].

IL-1β produced at the tumor site can induce macrophage chemotaxis. This was demonstrated in vitro with human metastatic melanoma samples and human cell lines [66], and in vivo in 3-methylcholanthrene (3-MCA)-induced skin cancer [68]. As shown in vitro with human gastric cancer cell lines, IL-1β increases macrophage recruitment by allowing monocyte chemoattractant protein (MCP)-1 expression by tumor cells [138].

Tumor cells can facilitate TAM-mediated IL-1β production. This was suggested by the fact that PDAC cell debris can stimulate IL-1β production by M2-polarized macrophages in vitro [139]. The sphingolipid sphingosine-1-phosphate (S1P), highly expressed by cancer cells, is able to trigger NLRP3 expression in macrophages and subsequent IL-1β production. This pathway plays a major role in tumor lymphangiogenesis, murine lymph node, and lung metastasis, while NLRP3 expression is correlated with human mammary carcinoma development [140,141]. Similarly, murine 4T1 breast cancer cells release soluble CD44, which in turn induces macrophage-mediated IL-1β production, leading to tumor growth and lung metastases [142]. Human lung cancer cells release microparticles that bind TLR3 to trigger NLRP3 inflammasome pathways in macrophages and IL-1β secretion. Thus, macrophages exposed to tumors may become inflammatory TAMs to promote human lung cancer development [143]. Moreover, PDAC cell exosomes alter macrophage phenotype and trigger inflammatory cytokine production, among which is IL-1β [144].

IL-1β derived from TAMs suppresses the expression of 15-hydroxyprostaglandin dehydrogenase (15-PGDH), an enzyme involved in prostaglandin degradation in PDAC cells, which results in tumor growth and poor prognosis for PDAC patients [145]. It can also increase COX-2 expression in human breast cancer cells, thus contributing to cancer progression [146]. In colon cancer, macrophage-derived IL-1β activates NF-κB-dependent PDK1/AKT signaling in tumor cells. This activates the Wnt pathway to enhance tumor growth [8,147]. In parallel, Wnt secretion by p53-deficient breast cancer cells activates IL-1β production by macrophages. IL-1β then activates neutrophils to dampen CD8-mediated anti-tumor immune response [122]. Finally, PDAC cells release ASC, which is able to act as an alarmin and induce IL-1β release by macrophages. Then, this IL-1β is able to trigger CAFs to release thymic stromal lymphopoietin (TSLP), which is a key cytokine for Th2 pro-tumor immune response [148].

Other factors can influence TAM polarization and inflammation. In a non-alcoholic fatty liver disease (NAFLD) model with colon cancer splenic xenograft, a high-fat diet induced TAM M2 polarization and substantial IL-1β and vascular endothelial growth factor (VEGF) production in an NLR family CARD containing 4 (NLRC4)-dependent manner. These events lead to increased liver metastasis, which can be countered using IL-1RA [149]. A high cholesterol diet is also responsible for macrophage production of IL-1β, through NLRP3 activation, and tumor growth in azoxymethane-induced colon cancer [150]. In the same context, obesity can be responsible for the pathogenesis of breast cancer. Human and murine breast tissue-associated adipocytes secrete C-C motif chemokine ligand 2 (CCL2) and IL-1β, which will both recruit and activate macrophages. These recruited cells secrete CXCL12, which is responsible for stromal vascularization and angiogenesis even before cancer occurrence [151]. Finally, in the lung, commensal bacteria stimulate Myd88-dependent IL-1β and IL-23 production from resident macrophages, inducing proliferation and activation of γδ T-cells that produce effector molecules (e.g., IL-17) to promote inflammation and tumor cell proliferation [152].

#### 3.2.3. Dendritic Cells

Dendritic cells (DCs) belong to the myeloid lineage. As the principle antigen-presenting cells of the immune system, DCs are immune sentinels and initiate T-cell response against microbial pathogens, tumors, and inflammation [153,154]. The use of DCs as cellular vaccines for immunotherapy has been studied for a long time. It consists in differentiating monocytes into DCs in vitro (with GM-CSF and IL-4). Many studies have tested the addition of a cytokine cocktail to improve DC maturation and activation. These cocktails are generally composed of IL-1β with IL-6, TNFα, and prostaglandin E2 (PGE2) [155,156].

Cancer cell-derived DAMPs (danger-associated molecular patterns) [157], double-stranded oligodeoxynucleotides [158], or bacteria [159,160] can be used to activate TLRs and the inflammasome to enable DCs to release IL-1β. For example, *Salmonella typhimurium* i.v. injected into mice can enable DCs to produce IL-1β and enhance inhibition of colon cancer growth. Inhibiting IL-1β restores tumor growth, while local administration of recombinant IL-1β inhibits tumor growth [159,160].

More physiologically, TMEM176B (transmembrane protein 176B, an immunoregulatory cation channel) has been identified as a new regulator of IL-1β production. In tumor-bearing mice, its deficiency leads to increased activation of caspase-1 and IL-1β production by DCs. In this context, IL-1β enhanced CD4^+^TCRβ^+^RORγt^+^ cells producing IL-17, which are responsible for slowing down tumor growth [161].

#### 3.2.4. Neutrophils

Neutrophils originate from myeloid precursors. Because of their phenotypic heterogeneity and functional versatility, neutrophils play a pivotal role in chronic inflammatory diseases, including cancer. Like macrophages, they can have anti- and pro-tumor functions in the tumor microenvironment [162]. Moreover, mature neutrophils share similar morphology and expression of cell surface markers with PMN-MDSCs, but the difference between these cell types relies on the suppression capacity of T-lymphocytes by PMN-MDSCs [163].

In an azoxymethane (AOM)/ dextran sodium sulfate (DSS)-induced cancer-associated colitis model, complement deficiency was shown to inhibit intestinal IL-1β production by neutrophils and IL-17A production by myeloid cells, and to repress tumor formation [164]. As shown in the 3-methylcholanthrene (3-MCA)-induced skin cancer model, IL-1β-deficient animals have fewer intra-tumor neutrophils, while IL-1RA-deficient mice have dense infiltrate, suggesting that IL-1β produced in tumors can recruit neutrophils [68].

However, the signaling of IL-1β in neutrophils can lead to opposite effects. In p53^-/-^ breast cancers, IL-1β-activated neutrophils curb CD8-mediated anti-tumor immune response [122]. In Epstein–Barr virus (EBV)-associated nasopharyngeal carcinoma, viral DNA and intra-tumor DAMPs stimulate inflammasomes to produce IL-1β. This low-level IL-1β favors tumor growth. On the contrary, treatment by irradiation or cisplatin increases tumor cell production of IL-1β, which recruits neutrophils. These tumor-associated neutrophils inhibit tumor growth [26]. In colorectal cancer, IL-1β may improve the control of local microbiota populations by neutrophils. This leads to a selection of microbe species, thus avoiding excessive pro-tumorigenic inflammatory cytokine production [70].

#### 3.2.5. T Lymphocytes

The adaptive immune response to cancer is regulated by T lymphocytes [165]. However, tumor-infiltrating CD4^+^ and CD8^+^ T-cells are associated with varying patient survival and clinical outcomes in many types of cancer such as breast [166], colorectal [167], and lung cancers [168]. CD4^+^ T-cell differentiation and CD8^+^ T-cell activation can be modulated by a cytokine network [169]. Among these cytokines is IL-1β.

Using a tetracycline-regulated human *IL1B* transgene in the mouse prostate, it was shown that IL-1β is able to induce the recruitment of CD4^+^ T-cells in inflammatory areas [170]. However, IL-1β has opposing effects on lymphocytes. One hypothesis to explain these discrepancies is the kinetics, frequency, and quantity of IL-1β. In different tumor models, it was shown that IL-1β injection may or may not decrease tumor growth, depending on the setting of the experiment. Injecting too early, or a single injection of IL-1β, has no effect on tumor growth, while several injections and higher doses (10 µg) inhibit tumor growth [171]. When these experiments are performed in immunodeficient mice, IL-1β has no effect, suggesting that T-cells participate in IL-1β-mediated effects.

This gives rise to the second possible explanation for the divergent effects of IL-1β; that is, perhaps it relies on the T cell subtypes present in the tumor. IL-1β seems to be required for secretion of Th1-derived cytokines IL-2 and IFN-γ at the tumor site, and subsequent blockade of B-cell myeloma and lymphoma growth [172]. The importance of IL-1β was confirmed by invalidating IL-1R1 in T-cells (it also inhibits IL-1α signaling). Thus, IL-1R1 signaling in T-cells entails Rorc expression and IL-17A and IL-22 production (suggesting a contribution of Th17 or innate lymphoid cells) and colon cancer progression [70,173,174]. Moreover, cancer cells and APCs from human ovarian cancer samples produce IL-1β, which favors the differentiation and expansion of Th17 cells [175]. Indirectly, IL-1β influences the fate of Treg cells. When produced by CAFs, it favors CCL22 production by tumor cells. CCL22 in turn allows recruitment and polarization of Tregs (through C-C motif chemokine receptor 4 (CCR4)-mediated forkhead box P3 (FOXP3) induction), responsible for the inhibition of the T-cell antitumor effect [176]. On the contrary, IL-1β induces IRF1 expression through STAT1, which then enables enhanced production of IL-9 and IL-21 in CD4 T-cells differentiated into Th9 cells. Th9 cells were shown to have anti-tumor properties. Consequently, Th9 cells generated in the presence of IL-1β exert more marked tumor inhibitory functions [177].

Tumor-derived IL1β activates γδT-cells to produce IL-17. Increased IL-17 levels lead to neutrophil expansion and alteration of their phenotype. These phenotypically altered neutrophils produce inducible nitric oxide synthase (iNOS), which inhibits the activity of anti-tumor CD8^+^ T-cells, resulting in an increase in the capacity of cancer cells to form metastases [178].

IL-1β was shown to have an effect on CD8^+^ T-cells. First, CD137L-mediated DC maturation leads to them producing IL-1β. Then, this cytokine leads to maturation of CD8^+^ T-cells, namely by increasing IFNγ and granzym B production [179,180]. Moreover, CD137L-maturated DC can also polarize CD8^+^ T-cells into Tc1 cells with less expression of exhaustion markers (CTLA-4, TIM-3, PD-1), but without showing the real impact of IL-1β in this phenotype modification [179]. In another study, IL-1β was shown to increase the proportion and functionality of adoptively transferred T-cells in the tumor and to lead to the inhibition of B16 melanoma tumor growth in mice. In this context, IL-1β increases trafficking and survival in peripheral tissues (lymph nodes, liver) and acts indirectly, through IL-15-dependent induction of Granzyme B production [181]. The effects of IL-1β on CD8^+^ T-cells should be further explored to define its mechanism of action and the molecular consequences on CD8^+^ phenotype, activity, and exhaustion.

IL-1β has different effects on immune cells. Thus, its pro- or anti-tumor effect may rely on the type and frequency of immune cells in the tumor.

### 3.3. Effects of IL-1β on Angiogenesis

Angiogenesis is a process that enables the formation of new blood vessels to support the growth of malignant tumors by supplying oxygen and nutrients to cancer cells [182]. This phenomenon is induced by HIF, which promotes oncogene activation, pro-angiogenic factor expression, and anti-angiogenic factor suppression. The most important pro-angiogenic factors are vascular endothelial growth factor (VEGF), platelet-derived growth factor (PDGF), and fibroblast growth factors (FGF) [183]. However, IL-1β is also an important regulator of angiogenesis.

In vitro, human samples (metastatic melanoma) and human cell lines (melanoma, oral squamous cell carcinoma (OSCC)) have been shown to produce IL-1β, which favors tube formation by HUVEC cells [66,89].

The impact of IL-1β on angiogenesis has also been observed in different cancer models. For example, in a transgenic model of Myc-dependent carcinogenesis, IL-1β triggers VEGF production and neo-angiogenesis [184]. Moreover, when experiments are performed in IL-1β-deficient mice or using IL-1RA, the vascularization of the tumor was abrogated [67,185,186]. This pro-angiogenic role was observed even when IL-1β was produced by different cell types, that is, cancer cells or myeloid cells. Fibrosarcoma or Lewis lung carcinoma (LLC) cells modified to constitutively secrete IL-1β were shown in vivo to promote angiogenesis, through the induction of VEGF, CXCL2, and hepatocyte growth factor production by cancer and stromal cells, leading to tumor progression [68,187,188]. In melanoma, production of VEGF and other proangiogenic factors by endothelial cells is dependent on myeloid cell (macrophages or MDSCs)-derived IL-1β [185,189].

In a mouse model with a high fat diet, obesity was shown to drive angiogenesis and cancer progression. Two mechanisms were proposed. Macrophage-derived IL-1β is able to stimulate the production of Angiopoietin-like 4 (ANGPTL4), a pro-angiogenic factor, in adipocytes [190]. NLRC4 inflammasome activation in myeloid cells can trigger the secretion of IL-1β, which in turn stimulates adipocytes to secrete VEGF-A [191].

### 3.4. Effects of IL-1β on Cancer Metastasis

Metastasis is the mechanism leading to the spread of cancer cells from the original site of the tumor to other major organs, such as the lung, liver, and kidney. It proceeds directly through invasion into the adjacent tissues or indirectly through several steps, including intravasation, circulation through blood or lymphatic vessels, anchoring at a secondary site, extravasation, and establishment of metastatic lesions in distant organs [192].

The importance of IL-1β in metastasis was first observed 30 years ago. Injection of IL-1β in mice increased lung and hepatic metastasis [193,194]. Moreover, IL-1β-overexpressing fibrosarcoma cells have an increased invasion potential [131,195]. On the contrary, the invalidation of IL-1β or inflammasome components, responsible for IL-1β maturation, was associated with reduced lung or hepatic metastasis [196,197]. Similar results were observed for breast cancer bone metastasis, using anakinra (IL-1RA) [198]. Moreover, IL-1β silencing decreases metastatic potential of murine prostate cancer cells, while its overexpression increases it [199].

IL-1β is able to regulate metastasis at various levels, for example, by regulating (EMT), cancer cell stemness, sphere formation, or migration/invasion.

EMT is the process responsible for cancer cells acquiring stem-like properties, as well as migratory and invasive capacities. However, inhibition of EMT also induces cancer stemness and mesenchymal–epithelial transition, the reverse process of EMT, which is associated with the tumor-initiating ability required for metastatic colonization [200]. This may explain observations relating to the role of IL-1β on EMT. On one hand, continuous exposure of NSCLC to IL-1β induces the EMT phenotype, with high expression of the transcription factor SLUG required for the establishment of EMT memory. Furthermore, even when IL-1β exposure was withdrawn, cancer cells sustained their acquired phenotype [115,201]. In breast cancer, IL-1β induces BIRC3 expression and estrogen receptor (ER) α gene methylation, leading to EMT [202,203]. IL-1β also stabilizes Snail, an EMT actor, in an NF-κB/AKT/Wnt-dependent manner in human colon cancer cells [204]. Anti-IL-1β antibodies, just like anti-IL-6, attenuated EMT phenotype in breast cancer cells [80]. On the other hand, at the metastatic site, IL-1β maintains cancer cells in a ZEB1-positive differentiation state, preventing their establishment. The absence of inflammation or blocking IL-1R removes the differentiation block and allows metastatic colonization. Among lymph node-positive breast cancer patients, high IL-1β expression in the primary tumor is associated with better overall survival and distant-metastasis-free survival [205]. These discrepancies in IL-1β activity need to be carefully considered when developing anti-IL-1β therapies.

Recombinant IL-1β enhances the sphere-forming capacity of cancer stem cells (CSCs) by increasing stemness gene expression (Bmi1 and Nestin) [206]. Furthermore, carcinoma-derived IL-1 (IL-1α and IL-1β) favors a transition from tumor cells into CSCs [207]. This is because of the capacity of IL-1 to allow mesenchymal stem cells to produce factors (PGE2, IL-6, IL-8, Gro-α, RANTES) that in turn activate β-catenin in cancer cells. β-catenin is a master regulator of proliferation, migration, and invasion [207]. IL-1β can also act directly on gastric cancer cells and induces PI3K activation and translocation of S100A4, a factor known to be involved in the metastasis of several types of cancer [208,209]. Finally, IL-1β-induced β1-integrin expression is responsible for ovarian tumor cell adhesion to mesothelia, a crucial step in ovarian cancer dissemination [210].

The IL-1β responsible for migration/invasion was shown to be produced by cells in the tumor microenvironment, such as macrophages, fibroblasts, and B-cells. In co-cultures of glioblastoma cells with PBMC, anakinra was shown to inhibit inflammatory crosstalk and cancer cell migration [211]. NLRP3 expression in TAMs is correlated with lymph node invasion, metastasis, and survival in mammary carcinoma patients [140]. This was sustained by the fact that inhibition or invalidation of NLRP3 in macrophages inhibited the metastatic potential of B16F10 murine melanoma cells in vitro [212]. Finally, macrophage-derived IL-1β was shown to regulate breast carcinoma cell migration and their adhesion to, and transmigration across, blood and lymphatic endothelial cells [213]. Cancer cells and fibroblasts can interact with each other to regulate cancer cell migration. First, tumor-induced tissue damage can be sensed as a DAMP by CAFs. This allows the activation of the NLRP3 inflammasome, the production of IL-1β, leading to tumor progression and lung metastasis [214]. Another study showed that IL-1β expressed in OSCC cells leads to CXCL1 production by CAFs, which in turn promotes cancer cell migration [215]. Further studies are required to evaluate whether these two pathways can co-exist. B-cells are more easily recruited in renal carcinoma tissues than in normal renal tissues. The interaction between B-cells and cancer cells allows IL-1β secretion, which is responsible for renal cancer cell migration, through HIF-2α and Notch1 pathways [216]. Production of IL-1β by cancer cells or neighboring cells can activate many molecular pathways that lead to cancer cell migration. It activates p38 in gastric cancer [84,217], extracellular signal-regulated protein kinase (ERK)1/2, AP-1, and MMP9 in invasive breast ductal carcinoma [218], and PI3K/Rac 1-regulated reorganization of the actin cytoskeleton of mammary MCF-7 cells [219]. IL-1β also increases TWIST expression in gallbladder cancer [220], and c-MYC, CCDN1, SNAIL1, and MMP2 expression through β-catenin pathway activation [221].

### 3.5. Pro- and Anti-Tumor Effects of IL-1β during Cancer Treatment

**Figure 3:**
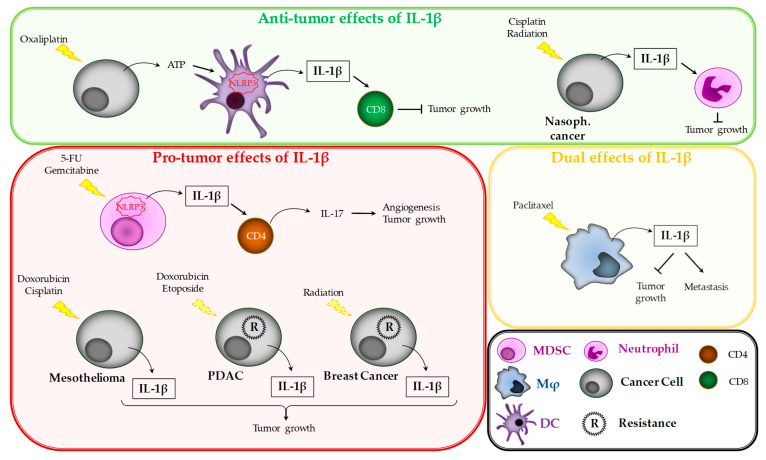
Involvement of IL-1β in chemotherapy-mediated effects on immune cells. PDAC, pancreatic ductal adenocarcinoma.

Beyond regulating cancer appearance or progression, IL-1β can also influence anti-cancer treatments. In fact, chemotherapy and radiation can trigger the production of IL-1β by either cancer cells or tumor infiltrating cells, such as macrophages, DCs, or MDSCs (Figure 3).

Chemotherapeutic agents such as doxorubicin or cisplatin give rise to NLRP3 expression, caspase-1 activation, and pyroptotic cell death of multiple mesothelioma cells. This leads to IL-1β release from cancer cells, and the use of anakinra in combination with cisplatin was shown to achieve decreased tumor growth in mice [222]. On the other hand, in nasopharyngeal carcinoma cells, the inflammasome is activated by cisplatin or radiation, through cathepsin B release from lysosomes or ROS production and mitochondrial DNA release into the cytosol, respectively. In this case, tumor-released IL-1β helps therapeutic treatments to inhibit tumor growth by recruiting neutrophils at the tumor site [26]. Chemoresistant cancer cells can also release IL-1β. This is the case of PDAC cell lines resistant to etoposide or doxorubicin, which constitutively secrete IL-1β, maintaining an NF-κB amplification loop responsible for chemoresistance [223,224]. This observation was confirmed on tumor samples that highly express p65 NF-κB subunit and IL-1β, contrary to normal pancreatic tissues [225,226]. Similarly, radiotherapy-resistant breast cancer cells secrete ATP, which in turn associates with its receptor P2Y2R on the cancer cell surface to induce caspase-1 activation and IL-1β release. Then, IL-1β induces MMP9 expression and invasion [227].

Chemotherapy can have opposing effects on anti-tumor immune response. First, it has been shown that anthracyclines, such as oxaliplatin, can activate NLRP3 inflammasome indirectly in DCs. Indeed, these compounds induce immunogenic cell death of cancer cells that release DAMPs such as ATP. Then, the released ATP associates with its receptor P2RX7 on DCs and induces caspase-1 activation and IL-1β release. The IL-1β thus released activates IFNγ-producing CD8+ T-cells [228]. In this context, the P2RX7/NLRP3 pathway is essential, as the anti-tumor effect of oxaliplatin is lost in mice deficient in these proteins. Moreover, breast cancer patients with a loss-of-function allele of P2RX7 developed more metastases than patients bearing the normal allele [228]. IL-1β inhibitors were also shown to reduce the anti-tumorigenic effect of oxaliplatin or anthracyclines [229]. On the contrary, we showed that other chemotherapies, such as 5-fluorouracil (5-FU) and gemcitabine, directly activate NLRP3 inflammasome in MDSCs, through a cathepsin B-dependent pathway, and enable IL-1β release by these cells. However, in this context, IL-1β targets CD4^+^ T-cells, which then produce IL-17. IL-17 is in turn responsible for neo-angiogenesis and tumor growth [230]. The NLRP3/IL-1β pathway is required for the deleterious effects of 5-FU and gemcitabine on tumor immune response, as tumor growth is inhibited in mice deficient for NLRP3, or IL-1R. Moreover, anakinra in combination with 5-FU inhibits tumor growth in mice and also enables stabilization of disease in refractory metastatic colorectal cancer patients, suggesting that this combination might have promise as a potential treatment [230,231]. We previously showed that HSP70 can inhibit NLRP3 inflammasome [232,233]. The importance of IL-1β in tumor escape from 5-FU treatment was strengthened, with the confirmation that HSP70 deficiency in mice leads to high caspase-1 activation in MDSCs, subsequent angiogenesis, and rapid tumor growth, whereas hyperthermia (which increases HSP70 expression) inhibits these events and slows down tumor growth [234].

Other chemotherapeutic agents have been shown to be capable of inducing IL-1β release from myeloid cells. For example, BRAF inhibitors vemurafenib and dabrafenib were shown to enable IL-1β secretion by human and murine DCs, but the authors did not explain whether this action on IL-1β was pro- or anti-tumor [114]. Paclitaxel favors NLRP3 activation in macrophages [235,236]. In different murine cancer types, this placlitaxel-induced macrophage IL-1β secretion slightly reduced the primary tumor, while promoting metastasis, suggesting a dual role for this drug [237].

These observations suggest that IL-1β may alternatively favor or inhibit chemotherapy-mediated anti-tumor immune response. Thus, the association of IL-1β or anti-IL-1β with chemotherapy should be considered, according to the drugs used.

## 4. Therapeutic Perspectives

We have seen that IL-1β is generally a promoter of cancer by acting on cancer cell proliferation and invasion, neo-angiogenesis, or tumor infiltrating immune cells. However, depending on the cancer type or stage, the main type of immune cells present in the tumor microenvironment, and the anti-cancer treatment used, inhibiting IL-1β may or may not be beneficial for patients.

IL-1β can be blocked at different levels: IL-1β itself, using antibodies, or IL-1β maturation, using inhibitors of inflammasomes, or inhibitors of the pathways leading to their activation.

Many antibodies have been developed to block IL-1β. The IL-1RA anakinra is one of the most widely used in pre-clinical studies. Anakinra is a non-glycosylated form of human IL-1RA that competitively inhibits IL-1α and IL-1β from binding to their receptor [238]. It has shown benefits in several clinical trials. Anakinra decreased the myeloma proliferative rate of smoldering or indolent multiple myeloma, leading to a chronic disease state and improved PFS [239]. In a phase II clinical study, we showed that using anakinra restored antitumor efficacy of 5-FU in heavily pretreated patients. Of the 32 patients enrolled, 5 showed a response (CHOI criteria) and 22 patients had stable disease [231]. Anakinra is currently being tested in further clinical trials. Another possible candidate is rilonacept, the extracellular domain of the IL-1RAcP and the IL-1R1 fused to the Fc portion of human IgG1. It has high affinity with IL-1β and IL-1α and potently inhibits IL-1 activity [240]. However, these blockers inhibit both IL-1β and IL-1α, but IL-1α can have tumor promoting or inhibiting functions, and inhibiting this isoform together with IL-1β can have synergistic or antagonist effects, depending on the context.

Canakinumab is a specific human monoclonal IgG1 antibody that targets IL-1β. This antibody has no cross-reactivity with either IL-1α or IL-1R1 [240]. A recent phase three clinical trial (CANTOS) involving 10,500 patients demonstrated that canakinumab could significantly reduce lung cancer incidence and patient mortality. However, fatal infections and sepsis were more common in the canakinumab group than in the placebo group [241]. Another mAb that inhibits IL-1β, gevokizumab, used in inflammatory disorders, could also be proposed in cancer treatment [242].

Several inflammasome chemical inhibitors tested in vivo and in vitro, such as MCC950, CY09, OLT1177, oridonin (targeting NLRP3 ATPase), or tranilast (targeting NLRP3 oligomerization), should be considered [243]. MCC950 was shown to successfully inhibit inflammation and to improve murine ulcerative colitis [244]. Specific caspase-1 inhibitors including ritonavir and VX-740/765 are also of interest. Ritonavir was originally developed for the treatment of HIV [245]. VX-765 is well tolerated and has shown benefits in a mouse model of rheumatoid arthritis [246]. Finally, we proposed hyperthermia as a new modulator of the NLRP3 inflammasome [232]. It can block caspase-1 activation in MDSCs, and subsequent angiogenesis and rapid tumor growth in mice [234]. Although hyperthermia is already used in specific treatment protocols, further studies are required to demonstrate its efficiency in humans. However, blocking inflammasomes may have limitations such as off-target effects, by inhibiting maturation of IL-18, an anti-tumor cytokine.

Another way to inhibit inflammasomes is to target molecular pathways leading to their activation. Thus, ion efflux (K^+^, Ca^2+^, Cl^−^), ROS or oxidized mitochondrial DNA generation, and lysosomal destabilization/cathepsin B can be targeted [247]. Potassium efflux can be inhibited by glyburide, a compound tested in gestational diabetes mellitus [248] or by P2RX7 inhibitors (oxATP, AZ10606120), which have shown anti-tumor effects in the murine B16 melanoma model [249]. ROS production can be dampened by antioxidants, which have shown health benefits when added to the diet. However, blocking one of these pathways requires adequate knowledge of its implication in cancer progression or resistance to treatment.

## 5. Conclusions

To conclude, we have highlighted in this review the pleiotropic effects of IL-1β in cancer. Although its role is primarily pro-tumoral, some examples have shown that it may also contribute to anti-tumor immune response. The exact explanations for these opposing effects are not yet known. The level of IL-1β produced, the type of producing cells, the microenvironment (immune cells or fibroblasts), the stage of the cancer, and the anti-cancer treatments used may all participate in the divergent effects of IL-1β. Further studies are required to elucidate these points. In any case, the use IL-1β blockers in the clinical context should be carefully considered, in order to guarantee the best treatment for patients.

## Figures and Tables

**Table 1:** Effects of interleukin (IL)-1β or IL1B expression or IL1B polymorphisms on cancer. NSCLC, non-small cell lung cancer.

Heading	Type of Cancer	Pro (+) or Anti (−) Tumor	Ref.
High IL-1β IHC staining	Nasopharyngeal carcinoma	(−)	[26]
High IL-1β blood level	NSCLC	(+)	[27,28,29]
High IL1B mRNA	Breast cancer	(−)	[30]
High IL1B mRNA	Cervical cancer	(−)	[31]
High IL1B-signature	Gliomas	(+)	[32]
*IL1B-511 C > T* (rs16944) T allele	Ovarian cancer	(+) or (−)	[33,34,35]
	Lung cancer	(+) or (−)	[36,37]
	Gastric cancer	(+) or (−)	[38,39,40,41,42,43,44,45,46]
	Cervical cancer	(+)	[47,48]
	Acute myeloid leukemia	(+)	[49]
	Chronic myeloid leukemia	(+)	[50]
*IL-1β-31 C > T* (rs1143627) T allele	Breast cancer	(+)	[51,52]
	Lung cancer	(+)	[36,37]
	Cervical cancer	(+)	[48]
	Hepatocellular carcinoma	(+)	[53]
	Osteosarcoma	(+)	[54]
*IL-1β-31 C > T* (rs1143627) C allele	Gastric cancer	(+)	[45]
*IL-1β-1464 G > C* (rs1143623) G allele	Renal cell carcinoma	(+)	[55]

## References

[1] Garlanda C., Dinarello C.A., Mantovani A. (2013). The interleukin-1 family: Back to the future. Immunity.

[2] Weber A., Wasiliew P., Kracht M. (2010). Interleukin-1 (IL-1) Processing Pathway. Sci. Signal..

[3] Rider P., Carmi Y., Voronov E., Apte R.N. (2013). Interleukin-1α. Semin. Immunol..

[4] Dinarello C.A. (2009). Immunological and Inflammatory Functions of the Interleukin-1 Family. Annu. Rev. Immunol..

[5] Schroder K., Tschopp J. (2010). The Inflammasomes. Cell.

[6] Bent R., Moll L., Grabbe S., Bros M. (2018). Interleukin-1 Beta—A Friend or Foe in Malignancies?. Int. J. Mol. Sci..

[7] Jarosz M., Olbert M., Wyszogrodzka G., Młyniec K., Librowski T. (2017). Antioxidant and anti-inflammatory effects of zinc. Zinc-dependent NF-κB signaling. Inflammopharmacology.

[8] Kaler P., Augenlicht L., Klampfer L. (2009). Macrophage-derived IL-1β stimulates Wnt signaling and growth of colon cancer cells: A crosstalk interrupted by vitamin D3. Oncogene.

[9] Afonina I.S., Müller C., Martin S.J., Beyaert R. (2015). Proteolytic Processing of Interleukin-1 Family Cytokines: Variations on a Common Theme. Immunity.

[10] Davis B.K., Wen H., Ting J.P.-Y. (2011). The inflammasome NLRs in immunity, inflammation, and associated diseases. Annu. Rev. Immunol..

[11] Kelley N., Jeltema D., Duan Y., He Y. (2019). The NLRP3 Inflammasome: An Overview of Mechanisms of Activation and Regulation. Int. J. Mol. Sci..

[12] Chevriaux A., Pilot T., Derangère V., Simonin H., Martine P., Chalmin F., Ghiringhelli F., Rébé C. (2020). Cathepsin B Is Required for NLRP3 Inflammasome Activation in Macrophages, Through NLRP3 Interaction. Front. Cell Dev. Boil..

[13] Sitia R., Rubartelli A. (2018). The unconventional secretion of IL-1β: Handling a dangerous weapon to optimize inflammatory responses. Semin. Cell Dev. Boil..

[14] Lopez-Castejon G., Brough D. (2011). Understanding the mechanism of IL-1β secretion. Cytokine Growth Factor Rev..

[15] Zhang M., Kenny S.J., Ge L., Xu K., Schekman R. (2015). Translocation of interleukin-1β into a vesicle intermediate in autophagy-mediated secretion. eLife.

[16] Qu Y., Franchi L., Nunez G., Dubyak G.R. (2007). Nonclassical IL-1β Secretion Stimulated by P2X7 Receptors Is Dependent on Inflammasome Activation and Correlated with Exosome Release in Murine Macrophages. J. Immunol..

[17] Monteleone M., Stanley A.C., Chen K.W., Brown D., Bezbradica J.S., von Pein J.B., Holley C.L., Boucher D., Shakespear M., Kapetanovic R. (2018). Interleukin-1β Maturation Triggers Its Relocation to the Plasma Membrane for Gasdermin-D-Dependent and -Independent Secretion. Cell Rep..

[18] Evavold C.L., Ruan J., Tan Y., Xia S., Wu H., Kagan J.C. (2018). The Pore-Forming Protein Gasdermin D Regulates Interleukin-1 Secretion from Living Macrophages. Immunity.

[19] Sborgi L., Rühl S., Mulvihill E., Pipercevic J., Heilig R., Stahlberg H., Farady C.J., Müller D.J., Brož P., Hiller S. (2016). GSDMD membrane pore formation constitutes the mechanism of pyroptotic cell death. EMBO J..

[20] Lachmann H.J., Lowe P., Felix S.D., Rordorf C., Leslie K., Madhoo S., Wittkowski H., Bek S., Hartmann N., Bosset S. (2009). In vivo regulation of interleukin 1β in patients with cryopyrin-associated periodic syndromes. J. Exp. Med..

[21] Cullen S.P., Kearney C.J., Clancy D.M., Martin S.J. (2015). Diverse Activators of the NLRP3 Inflammasome Promote IL-1β Secretion by Triggering Necrosis. Cell Rep..

[22] Mulay S.R., Kulkarni O.P., Rupanagudi K.V., Migliorini A., Darisipudi M.N., Vilaysane A., Muruve D., Shi Y., Munro F., Liapis H. (2012). Calcium oxalate crystals induce renal inflammation by NLRP3-mediated IL-1β secretion. J. Clin. Investig..

[23] Pelegrin P., Barroso-Gutierrez C., Surprenant A. (2008). P2X7 Receptor Differentially Couples to Distinct Release Pathways for IL-1β in Mouse Macrophage. J. Immunol..

[24] Garlanda C., Anders H.-J., Mantovani A. (2009). TIR8/SIGIRR: An IL-1R/TLR family member with regulatory functions in inflammation and T cell polarization. Trends Immunol..

[25] Loiarro M., Ruggiero V., Sette C. (2010). Targeting TLR/IL-1R Signalling in Human Diseases. Mediat. Inflamm..

[26] Chen L.-C., Wang L.-J., Tsang N.-M., Ojcius D.M., Chen C.-C., Ouyang C.-N., Hsueh C., Liang Y., Chang K.-P., Chen C.-C. (2012). Tumour inflammasome-derived IL-1β recruits neutrophils and improves local recurrence-free survival in EBV-induced nasopharyngeal carcinoma. EMBO Mol. Med..

[27] McLoed A.G., Sherrill T.P., Cheng N.-S., Han W., Saxon J.A., Gleaves L.A., Wu P., Polosukhin V.V., Karin M., Yull F.E. (2016). Neutrophil-Derived IL-1β Impairs the Efficacy of NF-κB Inhibitors against Lung Cancer. Cell Rep..

[28] Mitsunaga S., Ikeda M., Shimizu S., Ohno I., Furuse J., Inagaki M., Higashi S., Kato H., Terao K., Ochiai A. (2013). Serum levels of IL-6 and IL-1β can predict the efficacy of gemcitabine in patients with advanced pancreatic cancer. Br. J. Cancer.

[29] Kim J.-W., Koh Y., Kim N.-W., Ahn Y.-O., Kim T.M., Han S.-W., Oh -Y., Lee S.-H., Im S.-A., Kim T.-Y. (2013). Clinical Implications of VEGF, TGF-beta1, and IL-1beta in Patients with Advanced Non-small Cell Lung Cancer. Cancer Res. Treat..

[30] Martínez-Reza I., Díaz L., Barrera D., Segovia-Mendoza M., Pedraza-Sánchez S., Soca-Chafre G., Larrea F., García-Becerra R. (2019). Calcitriol Inhibits the Proliferation of Triple-Negative Breast Cancer Cells through a Mechanism Involving the Proinflammatory Cytokines IL-1βand TNF-α. J. Immunol. Res..

[31] Matamoros J.A., da Silva M.I.F., de Moura P.M.M.F., Leitão M.D.C.G., Coimbra E.C. (2019). Reduced Expression of IL-1β and IL-18 Proinflammatory Interleukins Increases the Risk of Developing Cervical Cancer. Asian Pac. J. Cancer Prev..

[32] Lee S.Y., Kim J.-K., Jeon H.-Y., Ham S.W., Kim H. (2017). CD133 Regulates IL-1β Signaling and Neutrophil Recruitment in Glioblastoma. Mol. Cells.

[33] Braicu I., Mustea A., Toliat M.R., Pirvulescu C., Könsgen D., Sun P., Nürnberg P., Lichtenegger W., Sehouli J. (2007). Polymorphism of IL-1α, IL-1β and IL-10 in patients with advanced ovarian cancer: Results of a prospective study with 147 patients. Gynecol. Oncol..

[34] Hefler L.A., Ludwig E., Lebrecht A., Zeillinger R., Tong-Cacsire D., Koelbl H., Leodolter S., Tempfer C.B. (2002). Polymorphisms of the interleukin-1 gene cluster and ovarian cancer. J. Soc. Gynecol. Investig..

[35] Ben-Ahmed A., Zidi S., Sghaier I., Ghazouani E., Mezlini A., Almawi W., Loueslati B.Y. (2017). Common variants in IL-1RN, IL-1β and TNF-α and the risk of ovarian cancer: A case control study. Central Eur. J. Immunol..

[36] Zienolddiny S., Ryberg D., Ms V.M., Skaug V., Canzian F., Haugen A. (2004). Polymorphisms of the interleukin-1? gene are associated with increased risk of non-small cell lung cancer. Int. J. Cancer.

[37] Bhat I.A., Naykoo N.A., Qasim I., Ganie F.A., Yousuf Q., Bhat B.A., Rasool R., Aziz S., Shah Z.A. (2014). Association of interleukin 1 beta (IL-1β) polymorphism with mRNA expression and risk of non small cell lung cancer. Meta Gene.

[38] El-Omar E.M., Carrington M., Chow W.-H., McColl K.E.L., Bream J.H., Young H.A., Herrera J., Lissowska J., Yuan C.-C., Rothman N. (2000). Interleukin-1 polymorphisms associated with increased risk of gastric cancer. Nature.

[39] Machado J.C., Pharoah P., Sousa S., Carvalho R., Oliveira C., Figueiredo C., Amorim A., Seruca R., Caldas C., Carneiro F. (2001). Interleukin 1B and interleukin 1RN polymorphisms are associated with increased risk of gastric carcinoma. Gastroenterology.

[40] Zeng Z.-R., Hu P.-J., Pang R.-P., Chen M.-H., Ng M., Sung J.J.Y. (2003). Association of interleukin 1B gene polymorphism and gastric cancers in high and low prevalence regions in China. Gut.

[41] Furuta T., El-Omar E., Xiao F., Shirai N., Takashima M., Sugimurra H. (2002). Interleukin 1β polymorphisms increase risk of hypochlorhydria and atrophic gastritis and reduce risk of duodenal ulcer recurrence in Japan. Gastroenterology.

[42] Camargo M.C. (2006). Interleukin-1 and Interleukin-1 Receptor Antagonist Gene Polymorphisms and Gastric Cancer: A Meta-analysis. Cancer Epidemiol. Biomark. Prev..

[43] Drici A.E.-M., Moulessehoul S., Tifrit A., Diaf M., Turki D.K., Bachir M., Tou A. (2016). Effect of IL-1β and IL-1RN polymorphisms in carcinogenesis of the gastric mucosa in patients infected with Helicobacter pylori in Algeria. Libyan J. Med..

[44] Chen B., Luo M.-X., Zhou X., Lv Y., Su G. (2016). Correlation Between Interleukin-1β-511 C/T Polymorphism and Gastric Cancer in Chinese Populations: A Meta-Analysis. Med Sci. Monit..

[45] He B.-S., Pan Y.-Q., Xu Y.-F., Zhu C., Qu L.-L., Wang S. (2011). Polymorphisms in Interleukin-1B (IL-1B) and Interleukin 1 Receptor Antagonist (IL-1RN) Genes Associate with Gastric Cancer Risk in the Chinese Population. Dig. Dis. Sci..

[46] Chang Y.-W., Jang J., Kim N.-H., Lee J.W., Lee H.J., Jung W.W., Dong S.-H., Kim H.-J., Kim B.-H., Lee J.-I. (2005). Interleukin-1B (IL-1B) polymorphisms and gastric mucosal levels of IL-1? Cytokine in Korean patients with gastric cancer. Int. J. Cancer.

[47] Lee Y.H., Song G.G. (2014). A meta-analysis of the association between CTLA-4 +49 A/G, −318 C/T, and IL-1 polymorphisms and susceptibility to cervical cancer. Neoplasma.

[48] Qian N., Chen X., Han S., Qiang F., Jin G., Zhou X., Dong J., Wang X., Shen H., Hu Z. (2009). Circulating IL-1β levels, polymorphisms of IL-1B, and risk of cervical cancer in Chinese women. J. Cancer Res. Clin. Oncol..

[49] Wang H., Hua M., Wang S., Yu J., Chen C., Zhao X., Zhang C., Zhong C., Wang R., He N. (2016). Genetic polymorphisms of IL-18 rs1946518 and IL-1β rs16944 are associated with prognosis and survival of acute myeloid leukemia. Inflamm. Res..

[50] Zhang A., Yu J., Yan S., Zhao X., Chen C., Zhou Y., Zhao X., Hua M., Wang R., Zhang C. (2018). The genetic polymorphism and expression profiles of NLRP3 inflammasome in patients with chronic myeloid leukemia. Hum. Immunol..

[51] Akisik E., Dalay N. (2007). Functional polymorphism of thymidylate synthase, but not of theCOMT andIL-1B genes, is associated with breast cancer. J. Clin. Lab. Anal..

[52] Eras N., Daloglu F.T., Çolak T., Guler M., Akbas E. (2019). The Correlation between IL-1β-C31T Gene Polymorphism and Susceptibility to Breast Cancer. J. Breast Cancer.

[53] Tak K.H., Yu G.I., Lee M.-Y., Shin D. (2018). Association Between Polymorphisms of Interleukin 1 Family Genes and Hepatocellular Carcinoma. Med. Sci. Monit..

[54] He Y., Liang X., Meng C., Shao Z., Gao Y., Wu Q., Liu J., Wang H., Yang S. (2014). Genetic polymorphisms of interleukin-1 beta and osteosarcoma risk. Int. Orthop..

[55] Wang F., Zhang Y., Wang S., Zhang Y., Wu D., Zhang C., Gao Y., Liu X., Wang W., Zhang S. (2017). IL1 genes polymorphism and the risk of renal cell carcinoma in Chinese Han population. Oncotarget.

[56] Chen M.-F., Lu M.S., Chen P.-T., Chen W.-C., Lin P.-Y., Lee K.-D. (2011). Role of interleukin 1 beta in esophageal squamous cell carcinoma. J. Mol. Med..

[57] Kai H., Kitadai Y., Kodama M., Cho S., Kuroda T., Ito M., Tanaka S., Ohmoto Y., Chayama K. (2005). Involvement of proinflammatory cytokines IL-1beta and IL-6 in progression of human gastric carcinoma. Anticancer. Res..

[58] Deans D.A.C., Wigmore S.J., Gilmour H., Paterson-Brown S., Ross J.A., Fearon K.C.H. (2006). Elevated tumour interleukin-1β is associated with systemic inflammation: A marker of reduced survival in gastro-oesophageal cancer. Br. J. Cancer.

[59] Al-Tahhan M.A., Etewa R.L., el Behery M.M. (2011). Association between circulating interleukin-1 beta (IL-1β) levels and IL-1β C–511T polymorphism with cervical cancer risk in Egyptian women. Mol. Cell. Biochem..

[60] El-Omar E., Carrington M., Chow W.-H., McColl K.E.L., Bream J.H., Young H.A., Herrera J., Lissowska J., Yuan C.-C., Rothman N. (2001). Correction: The role of interleukin-1 polymorphisms in the pathogenesis of gastric cancer. Nature.

[61] Fox J.G., Wang T.C. (2007). Inflammation, atrophy, and gastric cancer. J. Clin. Investig..

[62] Lind H., Haugen A., Zienolddiny S. (2007). Differential binding of proteins to the IL1B −31 T/C polymorphism in lung epithelial cells. Cytokine.

[63] Grimm C., Kantelhardt E.J., Heinze G., Polterauer S., Zeillinger R., Kölbl H., Reinthaller A., Hefler L. (2009). The prognostic value of four interleukin-1 gene polymorphisms in caucasian women with breast cancer—A multicenter study. BMC Cancer.

[64] Lee K.-A., Ki C.-S., Kim H.-J., Sohn K.-M., Kim J.-W., Kang W.K., Rhee J.C., Song S.Y., Sohn T.S. (2004). Novel interleukin 1β polymorphism increased the risk of gastric cancer in a Korean population. J. Gastroenterol..

[65] Zhong F.L., Mamaï O., Sborgi L., Boussofara L., Hopkins R., Robinson K., Szeverényi I., Takeichi T., Balaji R., Lau A. (2016). Germline NLRP1 Mutations Cause Skin Inflammatory and Cancer Susceptibility Syndromes via Inflammasome Activation. Cell.

[66] Okamoto M., Liu W., Luo Y., Tanaka A., Cai X., Norris D.A., Dinarello C.A., Fujita M. (2009). Constitutively Active Inflammasome in Human Melanoma Cells Mediating Autoinflammation via Caspase-1 Processing and Secretion of Interleukin-1β. J. Boil. Chem..

[67] Voronov E., Shouval D.S., Krelin Y., Cagnano E., Benharroch D., Iwakura Y., Dinarello C.A., Apte R.N. (2003). IL-1 is required for tumor invasiveness and angiogenesis. Proc. Natl. Acad. Sci. USA.

[68] Krelin Y., Voronov E., Dotan S., Elkabets M., Reich E., Fogel M., Huszar M., Iwakura Y., Segal S., Dinarello C.A. (2007). Interleukin-1β–Driven Inflammation Promotes the Development and Invasiveness of Chemical Carcinogen–Induced Tumors. Cancer Res..

[69] Chien C.-H., Lee M.-J., Liou H.-C., Liou H.-H., Fu W.-M. (2015). Local Immunosuppressive Microenvironment Enhances Migration of Melanoma Cells to Lungs in DJ-1 Knockout Mice. PLoS ONE.

[70] Dmitrieva-Posocco O., Dzutsev A., Posocco D.F., Hou V., Yuan W., Thovarai V., Mufazalov I.A., Gunzer M., Shilovskiy I., Khaitov M.R. (2019). Cell-Type-Specific Responses to Interleukin-1 Control Microbial Invasion and Tumor-Elicited Inflammation in Colorectal Cancer. Immunity.

[71] Allen I.C., TeKippe E.M., Woodford R.-M.T., Uronis J.M., Holl E.K., Rogers A.B., Herfarth H.H., Jobin C., Ting J.P.-Y. (2010). The NLRP3 inflammasome functions as a negative regulator of tumorigenesis during colitis-associated cancer. J. Exp. Med..

[72] Dupaul-Chicoine J., Yeretssian G., Doiron K., Bergstrom K.S., McIntire C.R., Leblanc P.M., Meunier C., Turbide C., Gros P., Beauchemin N. (2010). Control of Intestinal Homeostasis, Colitis, and Colitis-Associated Colorectal Cancer by the Inflammatory Caspases. Immunity.

[73] Zaki H., Boyd K.L., Vogel P., Kastan M.B., Lamkanfi M., Kanneganti T.-D. (2010). The NLRP3 Inflammasome Protects against Loss of Epithelial Integrity and Mortality during Experimental Colitis. Immunity.

[74] Ping P.H., Bo T.F., Li L., Hui Y.N., Zhu H. (2016). IL-1β/NF-kb signaling promotes colorectal cancer cell growth through miR-181a/PTEN axis. Arch. Biochem. Biophys..

[75] Matanic D., Beg-Zec Z., Stojanović D., Matakorić N., Flego V., Milevoj-Ribic F. (2003). Cytokines in Patients with Lung Cancer. Scand. J. Immunol..

[76] Wang L., Zhang L.-F., Wu J., Xu S., Xu Y.-Y., Li D., Lou J., Liu M.-F. (2014). IL-1β-Mediated Repression of microRNA-101 Is Crucial for Inflammation-Promoted Lung Tumorigenesis. Cancer Res..

[77] Jin L., Yuan R.Q., Fuchs A., Yao Y., Joseph A., Schwall R., Schnitt S.J., Guida A., Hastings H.M., Andres J. (1997). Expression of interleukin-1beta in human breast carcinoma. Cancer.

[78] Wu T., Hong Y., Jia L., Wu J., Xia J., Wang J., Hu Q., Cheng B. (2016). Modulation of IL-1β reprogrammes the tumor microenvironment to interrupt oral carcinogenesis. Sci. Rep..

[79] Snoussi K., Strosberg A.D., Bouaouina N., Ben-Ahmed S., Chouchane L. (2005). Genetic variation in pro-inflammatory cytokines (interleukin-1beta, interleukin-1alpha and interleukin-6) associated with the aggressive forms, survival, and relapse prediction of breast carcinoma. Eur. Cytokine Netw..

[80] Oh K., Lee O.-Y., Park Y., Seo M.W., Lee D.-S. (2016). IL-1β induces IL-6 production and increases invasiveness and estrogen-independent growth in a TG2-dependent manner in human breast cancer cells. BMC Cancer.

[81] Reed J.R., Leon R.P., Hall M.K., Schwertfeger K.L. (2009). Interleukin-1beta and fibroblast growth factor receptor 1 cooperate to induce cyclooxygenase-2 during early mammary tumourigenesis. Breast Cancer Res..

[82] Kaplanov I., Carmi Y., Kornetsky R., Shemesh A., Shurin G.V., Shurin M.R., Dinarello C.A., Voronov E., Apte R.N. (2018). Blocking IL-1β reverses the immunosuppression in mouse breast cancer and synergizes with anti–PD-1 for tumor abrogation. Proc. Natl. Acad. Sci. USA.

[83] Gomes T., Várady C.B.S., Lourenço A.L., Mizurini D.M., Rondon A.M.R., Leal A.C., Gonçalves B.S., Bou-Habib D.C., Medei E., Monteiro R.Q. (2019). IL-1β Blockade Attenuates Thrombosis in a Neutrophil Extracellular Trap-Dependent Breast Cancer Model. Front. Immunol..

[84] Tu S., Bhagat G., Cui G., Takaishi S., Kurt-Jones E.A., Rickman B., Betz K.S., Penz-Oesterreicher M., Bjorkdahl O., Fox J.G. (2008). Overexpression of Interleukin-1β Induces Gastric Inflammation and Cancer and Mobilizes Myeloid-Derived Suppressor Cells in Mice. Cancer Cell.

[85] Wu Y., Shen L., Liang X., Li S., Ma L., Zheng L., Li T., Yu H., Chan H., Chen C. (2019). Helicobacter pylori-induced YAP1 nuclear translocation promotes gastric carcinogenesis by enhancing IL-1β expression. Cancer Med..

[86] Yamanaka N., Morisaki T., Nakashima H., Tasaki A., Kubo M., Kuga H., Nakahara C., Nakamura K., Noshiro H., Yao T. (2004). Interleukin 1 Enhances Invasive Ability of Gastric Carcinoma through Nuclear Factor- B Activation. Clin. Cancer Res..

[87] Guo T., Qian J., Zhao Y.-Q., Li X.-B., Zhang J.-Z. (2012). Effects of IL-1? on the proliferation and apoptosis of gastric epithelial cells and acid secretion from isolated rabbit parietal cells. Mol. Med. Rep..

[88] Brailo V., Vucicevic-Boras V., Lukac J., Biocina-Lukenda D., Alajbeg I., Milenovic A., Balija M. (2011). Salivary and serum interleukin 1 beta, interleukin 6 and tumor necrosis factor alpha in patients with leukoplakia and oral cancer. Med. Oral Patol. Oral Cir. Bucal.

[89] Lee C.-H., Chang J.S.-M., Syu S.-H., Wong T.S., Chan J.Y.-W., Tang Y.-C., Yang Z.-P., Yang W.-C., Chen C.-T., Lu S.-C. (2014). IL-1β Promotes Malignant Transformation and Tumor Aggressiveness in Oral Cancer. J. Cell. Physiol..

[90] Zhang D., Li L., Jiang H., Li Q., Wang-Gillam A., Yu J., Head R., Liu J., Ruzinova M.B., Lim K.-H. (2018). Tumor–Stroma IL1β-IRAK4 Feedforward Circuitry Drives Tumor Fibrosis, Chemoresistance, and Poor Prognosis in Pancreatic Cancer. Cancer Res..

[91] Marrache F., Tu S.P., Bhagat G., Pendyala S., Österreicher C.H., Gordon S., Ramanathan V., Penz-Österreicher M., Betz K.S., Song Z. (2008). Overexpression of Interleukin-1β in the Murine Pancreas Results in Chronic Pancreatitis. Gastroenterology.

[92] Woolery K.T., Hoffman M.S., Kraft J., Nicosia S.V., Kumar A., Kruk P.A. (2014). Urinary interleukin-1β levels among gynecological patients. J. Ovarian Res..

[93] Stadlmann S., Pollheimer J., Moser P., Raggi A., Amberger A., Margreiter R., Offner F., Mikuz G., Dirnhofer S., Moch H. (2003). Cytokine-regulated expression of collagenase-2 (MMP-8) is involved in the progression of ovarian cancer. Eur. J. Cancer.

[94] Eiro N., Bermudez-Fernandez S., Fernández-García B., Atienza S., Beridze N., Escaf S., Vizoso F.J. (2014). Analysis of the Expression of Interleukins, Interferon β, and Nuclear Factor-κ B in Prostate Cancer and their Relationship With Biochemical Recurrence. J. Immunother..

[95] Rodríguez-Berriguete G., Sanchez-Espiridion B., Cansino J.R., Olmedilla G., Martínez-Onsurbe P., Sanchez-Chapado M., Paniagua R., Fraile B., Royuela M. (2013). Clinical significance of both tumor and stromal expression of components of the IL-1 and TNF-α signaling pathways in prostate cancer. Cytokine.

[96] Culig Z., Hobisch A., Herold M., Hittmair A., Thurnher M., Eder I., Cronauer M., Rieser C., Ramoner R., Bartsch G. (1998). Interleukin 1β mediates the modulatory effects of monocytes on LNCaP human prostate cancer cells. Br. J. Cancer.

[97] Albrecht M., Doroszewicz J., Gillen S., Gomes I., Wilhelm B., Stief T., Aumüller G. (2003). Proliferation of prostate cancer cells and activity of neutral endopeptidase is regulated by bombesin and IL-1β with IL-1β acting as a modulator of cellular differentiation. Prostate.

[98] Kawada M., Inoue H., Usami I., Takamoto K., Masuda T., Yamazaki Y., Ikeda D. (2006). Establishment of a highly tumorigenic LNCaP cell line having inflammatory cytokine resistance. Cancer Lett..

[99] Kawada M., Ishizuka M., Takeuchi T. (1999). Enhancement of Antiproliferative Effects of Interleukin-1β and Tumor Necrosis Factor-α on Human Prostate Cancer LNCaP Cells by Coculture with Normal Fibroblasts through Secreted Interleukin-6. Jpn. J. Cancer Res..

[100] Longoni N., Sarti M., Albino D., Civenni G., Malek A., Pinton S., Mello-Grand M., Ostano P., D’Ambrosio G., Sessa F. (2013). ETS Transcription Factor ESE1/ELF3 Orchestrates a Positive Feedback Loop That Constitutively Activates NF-?B and Drives Prostate Cancer Progression. Cancer Res..

[101] le Brun G., Aubin P., Soliman H., Ropiquet F., Villette J.-M., Berthon P., Créminon C., Cussenot O., Fiet J. (1999). Upregulation of endothelin 1 and its precursor by IL-1beta, TNF-alpha, and TGF-beta in the PC3 human prostate cancer cell line. Cytokine.

[102] Klein R.D., Borchers A.H., Sundareshan P., Bougelet C., Berkman M.R., Nagle R.B., Bowden G. (1997). Interleukin-1β Secreted from Monocytic Cells Induces the Expression of Matrilysin in the Prostatic Cell Line LNCaP. J. Boil. Chem..

[103] Thomas-Jardin S.E., Kanchwala M., Jacob J., Merchant S., Meade R., Gahnim N.M., Nawas A.F., Xing C., Delk N.A. (2018). Identification of an IL-1-induced gene expression pattern in AR+PCa cells that mimics the molecular phenotype of AR−PCa cells. Prostate.

[104] Chang M., Patel V., Gwede M., Morgado M., Tomasevich K., Fong E., Farach-Carson M.C., Delk N.A. (2014). IL-1β induces p62/SQSTM1 and represses androgen receptor expression in prostate cancer cells. J. Cell. Biochem..

[105] Shahriari K., Shen F., Worrede A., Liu Q., Gong Y., Garcia F.U., Fatatis A. (2016). Cooperation among heterogeneous prostate cancer cells in the bone metastatic niche. Oncogene.

[106] Salman H., Bergman M., Blumberger N., Djaldetti M., Bessler H. (2014). Do androgen deprivation drugs affect the immune crosstalk between mononuclear and prostate cancer cells?. Biomed. Pharmacother..

[107] Beaupre D.M., Talpaz M., Marini F.C., Cristiano R.J., A Roth J., Estrov Z., Albitar M., Freedman M.H., Kurzrock R. (1999). Autocrine interleukin-1beta production in leukemia: Evidence for the involvement of mutated RAS. Cancer Res..

[108] Hamarsheh S., Osswald L., Saller B.S., Unger S., de Feo D., Vinnakota J.M., Konantz M., Uhl F.M., Becker H., Lübbert M. (2020). Oncogenic KrasG12D causes myeloproliferation via NLRP3 inflammasome activation. Nat. Commun..

[109] Takahashi R., Macchini M., Sunagawa M., Jiang Z., Tanaka T., Valenti G., Renz B.W., White R.A., Hayakawa Y., Westphalen C.B. (2020). Interleukin-1beta-induced pancreatitis promotes pancreatic ductal adenocarcinoma via B lymphocyte-mediated immune suppression. Gut.

[110] Marazioti A., Lilis I., Vreka M., Apostolopoulou H., Kalogeropoulou A., Giopanou I., Giotopoulou G.A., Krontira A.C., Iliopoulou M., Kanellakis N.I. (2018). Myeloid-derived interleukin-1β drives oncogenic KRAS-NF-κΒ addiction in malignant pleural effusion. Nat. Commun..

[111] Khalili J.S., Liu S., Rodríguez-Cruz T.G., Whittington M., Wardell S., Liu C., Zhang M., Cooper Z.A., Frederick D.T., Li Y. (2012). Oncogenic BRAF(V600E) promotes stromal cell-mediated immunosuppression via induction of interleukin-1 in melanoma. Clin. Cancer Res..

[112] Whipple C.A., E Brinckerhoff C. (2014). BRAFV600E melanoma cells secrete factors that activate stromal fibroblasts and enhance tumourigenicity. Br. J. Cancer.

[113] Zhou D., Li Z., Bai X. (2018). BRAF V600E and RET/PTC Promote the Activity of Nuclear Factor-κB, Inflammatory Mediators, and Lymph Node Metastasis in Papillary Thyroid Carcinoma: A Study of 50 Patients in Inner Mongolia. Med Sci. Monit..

[114] Hajek E., Krebs F., Bent R., Haas K., Bast A., Steinmetz I., Tuettenberg A., Grabbe S., Bros M. (2018). BRAF inhibitors stimulate inflammasome activation and interleukin 1 beta production in dendritic cells. Oncotarget.

[115] Huang J., Lan X., Wang T., Lu H., Cao M., Yan S., Cui Y., Jia D., Cai L., Xing Y. (2019). Targeting the IL-1β/EHD1/TUBB3 axis overcomes resistance to EGFR-TKI in NSCLC. Oncogene.

[116] Ma J., Liu J., Wang Z.-M., Gu X., Fan Y., Zhang W., Xu L., Zhang J., Cai D. (2014). NF-kappaB-dependent MicroRNA-425 upregulation promotes gastric cancer cell growth by targeting PTEN upon IL-1β induction. Mol. Cancer.

[117] Wang W., Wang Z., Chen S., Zang X., Miao J. (2018). Interleukin-1β/nuclear factor-κB signaling promotes osteosarcoma cell growth through the microRNA-181b/phosphatase and tensin homolog axis. J. Cell. Biochem..

[118] Huang Y., Wang H., Hao Y., Lin H., Dong M., Ye J., Song L., Wang Y., Li Q., Shan B. (2020). Myeloid PTEN promotes chemotherapy-induced NLRP3-inflammasome activation and antitumour immunity. Nature.

[119] Schauer I.G., Zhang J., Xing Z., Guo X., Mercado-Uribe I., Sood A.K., Huang P., Liu J. (2013). Interleukin-1β Promotes Ovarian Tumorigenesis through a p53/NF-κB-Mediated Inflammatory Response in Stromal Fibroblasts. Neoplasia.

[120] Qin Y., Ekmekcioglu S., Liu P., Duncan L.M., Lizée G., Poindexter N., Grimm E.A. (2011). Constitutive aberrant endogenous interleukin-1 facilitates inflammation and growth in human melanoma. Mol. Cancer Res..

[121] Ubertini V., Norelli G., D’Arcangelo D., Gurtner A., Cesareo E., Baldari S., Gentileschi M.P., Piaggio G., Nisticò P., Soddu S. (2014). Mutant p53 gains new function in promoting inflammatory signals by repression of the secreted interleukin-1 receptor antagonist. Oncogene.

[122] Wellenstein M.D., Coffelt S.B., Duits D.E.M., van Miltenburg M.H., Slagter M., de Rink I., Henneman L., Kas S.M., Prekovic S., Hau C.-S. (2019). Loss of p53 triggers Wnt-dependent systemic inflammation to drive breast cancer metastasis. Nature.

[123] Vikhreva P., Petrova V., Gokbulut T., Pestlikis I., Mancini M., Di Daniele N., Knight R.A., Melino G., Amelio I. (2017). TAp73 upregulates IL-1β in cancer cells: Potential biomarker in lung and breast cancer?. Biochem. Biophys. Res. Commun..

[124] Woolery K.T., Mohamed M., Linger R.J., Dobrinski K.P., Roman J., Kruk P.A. (2015). BRCA1 185delAG Mutation Enhances Interleukin-1β Expression in Ovarian Surface Epithelial Cells. BioMed Res. Int..

[125] Dutta D., Dutta S., Veettil M.V., Roy A., Ansari M.A., Iqbal J., Chikoti L., Kumar B., Johnson K.E., Chandran B. (2015). BRCA1 Regulates IFI16 Mediated Nuclear Innate Sensing of Herpes Viral DNA and Subsequent Induction of the Innate Inflammasome and Interferon-β Responses. PLoS Pathog..

[126] Gabrilovich D.I., Bronte V., Chen S.H., Colombo M.P., Ochoa A., Ostrand-Rosenberg S., Schreiber H. (2007). The terminology issue for myeloid-derived suppressor cells. Cancer Res..

[127] Almand B., Clark J.I., Nikitina E., van Beynen J., English N.R., Knight S.C., Carbone D.P., Gabrilovich D.I. (2001). Increased production of immature myeloid cells in cancer patients: A mechanism of immunosuppression in cancer. J. Immunol..

[128] Díaz-Montero C.M., Salem M.L., Nishimura M.I., Garrett-Mayer E., Cole D.J., Montero A.J. (2008). Increased circulating myeloid-derived suppressor cells correlate with clinical cancer stage, metastatic tumor burden, and doxorubicin–cyclophosphamide chemotherapy. Cancer Immunol. Immunother..

[129] Nagaraj S., Gupta K., Pisarev V., Kinarsky L., Sherman S., Kang L., Herber D.L., Schneck J., Gabrilovich D.I. (2007). Altered recognition of antigen is a mechanism of CD8+ T cell tolerance in cancer. Nat. Med..

[130] Bunt S.K., Yang L., Sinha P., Clements V.K., Leips J., Ostrand-Rosenberg S. (2007). Reduced inflammation in the tumor microenvironment delays the accumulation of myeloid-derived suppressor cells and limits tumor progression. Cancer Res..

[131] Song X., Krelin Y., Dvorkin T., Bjorkdahl O., Segal S., Dinarello C.A., Voronov E., Apte R.N. (2005). CD11b+/Gr-1+ Immature Myeloid Cells Mediate Suppression of T Cells in Mice Bearing Tumors of IL-1β-Secreting Cells. J. Immunol..

[132] Bunt S.K., Sinha P., Clements V.K., Leips J., Ostrand-Rosenberg S. (2006). Inflammation induces myeloid-derived suppressor cells that facilitate tumor progression. J. Immunol..

[133] Elkabets M., Ribeiro V.S.G., Dinarello C.A., Ostrand-Rosenberg S., Di Santo J.P., Apte R.N., Vosshenrich C. (2010). IL-1β regulates a novel myeloid-derived suppressor cell subset that impairs NK cell development and function. Eur. J. Immunol..

[134] Bunt S.K., Clements V.K., Hanson E.M., Sinha P., Ostrand-Rosenberg S. (2009). Inflammation enhances myeloid-derived suppressor cell crosstalk by signaling through Toll-like receptor 4. J. Leukoc. Boil..

[135] Mills C.D., Kincaid K., Alt J.M., Heilman M.J., Hill A.M. (2000). M-1/M-2 macrophages and the Th1/Th2 paradigm. J. Immunol..

[136] Martinez F.O. (2008). Macrophage activation and polarization. Front. Biosci..

[137] Rey-Giraud F., Hafner M., Ries C.H. (2012). In Vitro Generation of Monocyte-Derived Macrophages under Serum-Free Conditions Improves Their Tumor Promoting Functions. PLoS ONE.

[138] Li S., Wang W., Zhang N., Ma T., Zhao C. (2014). IL-1β mediates MCP-1 induction by Wnt5a in gastric cancer cells. BMC Cancer.

[139] Chen Q., Wang J., Zhang Q., Zhang J., Lou Y., Yang J., Chen Y., Wei T., Zhang J., Fu Q. (2019). Tumour cell-derived debris and IgG synergistically promote metastasis of pancreatic cancer by inducing inflammation via tumour-associated macrophages. Br. J. Cancer.

[140] Weichand B., Popp R., Dziumbla S., Mora J., Strack E., Elwakeel E., Frank A.-C., Scholich K., Pierre S., Syed S.N. (2017). S1PR1 on tumor-associated macrophages promotes lymphangiogenesis and metastasis via NLRP3/IL-1β. J. Exp. Med..

[141] Watari K., Shibata T., Kawahara A., Sata K.-I., Nabeshima H., Shinoda A., Abe H., Azuma K., Murakami Y., Izumi H. (2014). Tumor-Derived Interleukin-1 Promotes Lymphangiogenesis and Lymph Node Metastasis through M2-Type Macrophages. PLoS ONE.

[142] Jang J.-H., Kim D.-H., Lim J.M., Lee J.W., Jeong S.J., Kim K.P., Dong Z. (2020). Breast cancer cell-derived soluble CD44 promotes tumor progression by triggering macrophage IL-1β production. Cancer Res..

[143] Chen J., Sun W., Zhang H., Ma J., Xu P., Yu Y., Fang H., Zhou L., Lv J., Xie J. (2019). Macrophages reprogrammed by lung cancer microparticles promote tumor development via release of IL-1beta. Cell Mol. Immunol..

[144] Linton S.S., Abraham T., Liao J., Clawson G.A., Butler P.J., Fox T., Kester M., Matters G.L. (2018). Tumor-promoting effects of pancreatic cancer cell exosomes on THP-1-derived macrophages. PLoS ONE.

[145] Arima K., Komohara Y., Bu L., Tsukamoto M., Itoyama R., Miyake K., Uchihara T., Ogata Y., Nakagawa S., Okabe H. (2018). Downregulation of 15-hydroxyprostaglandin dehydrogenase by interleukin-1β from activated macrophages leads to poor prognosis in pancreatic cancer. Cancer Sci..

[146] Hou Z., Falcone D.J., Subbaramaiah K., Dannenberg A.J. (2011). Macrophages induce COX-2 expression in breast cancer cells: Role of IL-1β autoamplification. Carcinogenesis.

[147] Kaler P., Godasi B.N., Augenlicht L., Klampfer L. (2009). The NF-κB/AKT-dependent Induction of Wnt Signaling in Colon Cancer Cells by Macrophages and IL-1β. Cancer Microenviron..

[148] Brunetto E., de Monte L., Balzano G., Camisa B., Laino V., Riba M., Heltai S., Bianchi M., Bordignon C., Falconi M. (2019). The IL-1/IL-1 receptor axis and tumor cell released inflammasome adaptor ASC are key regulators of TSLP secretion by cancer associated fibroblasts in pancreatic cancer. J. Immunother. Cancer.

[149] Ohashi K., Wang Z., Yang Y.M., Billet S., Tu W., Pimienta M., Cassel S.L., Pandol S.J., Lu S.C., Sutterwala F.S. (2019). NOD-like receptor C4 Inflammasome Regulates the Growth of Colon Cancer Liver Metastasis in NAFLD. Hepatology.

[150] Du Q., Wang Q., Fan H., Wang J., Liu X., Wang H., Wang Y., Hu R. (2016). Dietary cholesterol promotes AOM-induced colorectal cancer through activating the NLRP3 inflammasome. Biochem. Pharmacol..

[151] Arendt L.M., McCready J., Keller P.J., Baker D.D., Naber S.P., Seewaldt V., Kuperwasser C. (2013). Obesity promotes breast cancer by CCL2-mediated macrophage recruitment and angiogenesis. Cancer Res..

[152] Jin C., Lagoudas G.K., Zhao C., Bullman S., Bhutkar A., Hu B., Ameh S., Sandel D., Liang X.S., Mazzilli S. (2019). Commensal Microbiota Promote Lung Cancer Development via γδ T Cells. Cell.

[153] Crowley M., Inaba K., Steinman R.M. (1990). Dendritic cells are the principal cells in mouse spleen bearing immunogenic fragments of foreign proteins. J. Exp. Med..

[154] Steinman R.M. (1991). The Dendritic Cell System and its Role in Immunogenicity. Annu. Rev. Immunol..

[155] Wieckowski E., Chatta G.S., Mailliard R.M., Gooding W., Palucka K., Banchereau J., Kalinski P. (2010). Type-1 polarized dendritic cells loaded with apoptotic prostate cancer cells are potent inducers of CD8+ T cells against prostate cancer cells and defined prostate cancer-specific epitopes. Prostate.

[156] Peng J.C., Thomas R., Nielsen L.K. (2005). Generation and Maturation of Dendritic Cells for Clinical Application Under Serum-Free Conditions. J. Immunother..

[157] Fang H., Ang B., Xu X., Huang X., Wu Y., Sun Y., Wang W., Li N., Cao X., Wan T. (2013). TLR4 is essential for dendritic cell activation and anti-tumor T-cell response enhancement by DAMPs released from chemically stressed cancer cells. Cell. Mol. Immunol..

[158] Koo J.E., Shin S.W., Um S.H., Lee J.Y. (2015). X-shaped DNA potentiates therapeutic efficacy in colitis-associated colon cancer through dual activation of TLR9 and inflammasomes. Mol. Cancer.

[159] Wu Y., Feng Z., Jiang S., Chen J., Zhan Y., Chen J. (2019). Secreting-lux/pT-ClyA engineered bacteria suppresses tumor growth via interleukin-1β in two pathways. AMB Express.

[160] Kim J.-E., Phan T.X., Nguyen V.H., Dinh-Vu H.-V., Zheng J.H., Yun M., Park S.-G., Hong Y., Choy H.E., Szardenings M. (2015). Salmonella typhimurium Suppresses Tumor Growth via the Pro-Inflammatory Cytokine Interleukin-1β. Theranostics.

[161] Segovia M., Russo S., Jeldres M., Mahmoud Y., Perez V., Duhalde M., Charnet P., Rousset M., Victoria S., Veigas F. (2019). Targeting TMEM176B Enhances Antitumor Immunity and Augments the Efficacy of Immune Checkpoint Blockers by Unleashing Inflammasome Activation. Cancer Cell.

[162] Wu L., Saxena S., Awaji M., Singh R.K. (2019). Wu Tumor-Associated Neutrophils in Cancer: Going Pro. Cancers.

[163] Gabrilovich D.I., Nagaraj S. (2009). Myeloid-derived suppressor cells as regulators of the immune system. Nat. Rev. Immunol..

[164] Ning C., Wang Y., Han G.-C., Wang R., Xiao H., Li X.-Y., Hou C.-M., Ma Y.-F., Sheng D.-S., Shen B.-F. (2015). Complement activation promotes colitis-associated carcinogenesis through activating intestinal IL-1β/IL-17A axis. Mucosal Immunol..

[165] Shiku H. (2003). Importance of CD4+ helper T-cells in antitumor immunity. Int. J. Hematol..

[166] Marrogi A.J., Munshi A., Merogi A.J., Ohadike Y., El-Habashi A., Marrogi O.L., Freeman S.M. (1997). Study of tumor infiltrating lymphocytes and transforming growth factor-beta as prognostic factors in breast carcinoma. Int. J. Cancer.

[167] Galon J., Coleno-Costes A., Kirilovsky A., Mlecnik B., Lagorce-Pagès C., Tosolini M., Camus M., Zinzindohoué F., Bruneval P., Cugnenc P.-H. (2006). Type, Density, and Location of Immune Cells Within Human Colorectal Tumors Predict Clinical Outcome. Science.

[168] Hiraoka K., Miyamoto M., Cho Y., Suzuoki M., Oshikiri T., Nakakubo Y., Itoh T., Ohbuchi T., Kondo S., Katoh H. (2006). Concurrent infiltration by CD8+ T cells and CD4+ T cells is a favourable prognostic factor in non-small-cell lung carcinoma. Br. J. Cancer.

[169] Tüting T., Storkus W.J., Lotze M.T. (1997). Gene-based strategies for the immunotherapy of cancer. J. Mol. Med..

[170] Ashok A., Keener R.A., Rubenstein M., Stookey S., Bajpai S., Hicks J., Alme A.K., Drake C.G., Zheng Q., Trabzonlu L. (2019). Consequences of interleukin 1β-triggered chronic inflammation in the mouse prostate gland: Altered architecture associated with prolonged CD4+infiltration mimics human proliferative inflammatory atrophy. Prostate.

[171] North R.J., Neubauer R.H., Huang J.J., Newton R.C., E Loveless S. (1988). Interleukin 1-induced, T cell-mediated regression of immunogenic murine tumors. Requirement for an adequate level of already acquired host concomitant immunity. J. Exp. Med..

[172] Haabeth O.A.W., Lorvik K.B., Yagita H., Bogen B., Corthay A. (2015). Interleukin-1 is required for cancer eradication mediated by tumor-specific Th1 cells. OncoImmunology.

[173] Chung Y., Chang S.H., Martinez G.J., Yang X.O., Nurieva R., Kang H.S., Ma L., Watowich S.S., Jetten A.M., Tian Q. (2009). Critical Regulation of Early Th17 Cell Differentiation by Interleukin-1 Signaling. Immunity.

[174] Hu W., Troutman T.D., Edukulla R., Pasare C. (2011). Priming Microenvironments Dictate Cytokine Requirements for T Helper 17 Cell Lineage Commitment. Immunity.

[175] Miyahara Y., Odunsi K., Chen W., Peng G., Matsuzaki J., Wang R.-F. (2008). Generation, and regulation of human CD4+ IL-17-producing T cells in ovarian cancer. Proc. Natl. Acad. Sci. USA.

[176] Huang Y., Chang C., Kuo Y., Fang W., Kao H., Tsai S., Wu L.-W. (2019). Cancer-associated fibroblast-derived interleukin-1β activates protumor C-C motif chemokine ligand 22 signaling in head and neck cancer. Cancer Sci..

[177] Vegran F., Berger H., Boidot R., Mignot G., Bruchard M., Dosset M., Chalmin F., Rébé C., Derangere V., Ryffel B. (2014). The transcription factor IRF1 dictates the IL-21-dependent anticancer functions of TH9 cells. Nat. Immunol..

[178] Coffelt S.B., Kersten K., Doornebal C.W., Weiden J., Vrijland K., Hau C.-S., Verstegen N., Ciampricotti M., Hawinkels L.J., Jonkers J. (2015). IL-17-producing γδ T cells and neutrophils conspire to promote breast cancer metastasis. Nature.

[179] Dharmadhikari B., Nickles E., Harfuddin Z., Ishak N.D.B., Zeng Q., Bertoletti A., Schwarz H. (2018). CD137L dendritic cells induce potent response against cancer-associated viruses and polarize human CD8+ T cells to Tc1 phenotype. Cancer Immunol. Immunother..

[180] Ben-Sasson S.Z., Hogg A., Hu-Li J., Wingfield P., Chen X., Crank M., Caucheteux S., Ratner-Hurevich M., Berzofsky J.A., Nir-Paz R. (2013). IL-1 enhances expansion, effector function, tissue localization, and memory response of antigen-specific CD8 T cells. J. Exp. Med..

[181] Lee P.-H., Yamamoto T.N., Gurusamy D., Sukumar M., Yu Z., Hu-Li J., Kawabe T., Gangaplara A., Kishton R.J., Henning A.N. (2019). Host conditioning with IL-1β improves the antitumor function of adoptively transferred T cells. J. Exp. Med..

[182] Voronov E., Carmi Y., Apte R.N. (2014). The role IL-1 in tumor-mediated angiogenesis. Front. Physiol..

[183] Saravanan S., Vimalraj S., Pavani K., Ramesh N., Sumantran V.N. (2020). Intussusceptive angiogenesis as a key therapeutic target for cancer therapy. Life Sci.

[184] Shchors K., Shchors E., Rostker F., Lawlor E.R., Brown-Swigart L., Evan G.I. (2006). The Myc-dependent angiogenic switch in tumors is mediated by interleukin 1β. Genome Res..

[185] Carmi Y., Voronov E., Dotan S., Lahat N., Rahat M.A., Fogel M., Huszar M., White M.R., Dinarello C.A., Apte R.N. (2009). The Role of Macrophage-Derived IL-1 in Induction and Maintenance of Angiogenesis. J. Immunol..

[186] Bar D., Apte R.N., Voronov E., Dinarello C.A., Cohen S. (2003). A continuous delivery system of IL-1 receptor antagonist reduces angiogenesis and inhibits tumor development. FASEB J..

[187] Saijo Y., Tanaka M., Miki M., Usui K., Suzuki T., Maemondo M., Hong X., Tazawa R., Kikuchi T., Matsushima K. (2002). Proinflammatory Cytokine IL-1β Promotes Tumor Growth of Lewis Lung Carcinoma by Induction of Angiogenic Factors: In Vivo Analysis of Tumor-Stromal Interaction. J. Immunol..

[188] Nakao S., Kuwano T., Tsutsumi-Miyahara C., Ueda S.-I., Kimura Y.N., Hamano S., Sonoda K.-H., Saijo Y., Nukiwa T., Strieter R.M. (2005). Infiltration of COX-2–expressing macrophages is a prerequisite for IL-1β–induced neovascularization and tumor growth. J. Clin. Investig..

[189] Carmi Y., Dotan S., Rider P., Kaplanov I., White M.R., Baron R., Abutbul S., Huszar M., Dinarello C.A., Apte R.N. (2013). The Role of IL-1β in the Early Tumor Cell–Induced Angiogenic Response. J. Immunol..

[190] Kolb R., Kluz P., Tan Z.W., Borcherding N., Bormann N., Vishwakarma A., Balcziak L., Zhu P., Davies B.S., Gourronc F. (2018). Obesity-associated inflammation promotes angiogenesis and breast cancer via angiopoietin-like 4. Oncogene.

[191] Kolb R., Phan L., Borcherding N., Liu Y., Yuan F., Janowski A.M., Xie Q., Markan K.R., Li W., Potthoff M.J. (2016). Obesity-associated NLRC4 inflammasome activation drives breast cancer progression. Nat. Commun..

[192] Amin M.K.B.A., Shimizu A., Ogita H., Amin A. (2019). The Pivotal Roles of the Epithelial Membrane Protein Family in Cancer Invasiveness and Metastasis. Cancers.

[193] Giavazzi R., Garofalo A., Bani M.R., Abbate M., Ghezzi P., Boraschi D., Mantovani A., Dejana E. (1990). Interleukin 1-induced augmentation of experimental metastases from a human melanoma in nude mice. Cancer Res..

[194] Vidal-Vanaclocha F., Amézaga C., Asumendi A., Kaplanski G., A Dinarello C. (1994). Interleukin-1 receptor blockade reduces the number and size of murine B16 melanoma hepatic metastases. Cancer Res..

[195] Song X., Voronov E., Dvorkin T., Fima E., Cagnano E., Benharroch D., Shendler Y., Bjorkdahl O., Segal S., Dinarello C.A. (2003). Differential Effects of IL-1α and IL-1β on Tumorigenicity Patterns and Invasiveness. J. Immunol..

[196] Vidal-Vanaclocha F., Fantuzzi G., Mendoza L., Fuentes A.M., Anasagasti M.J., Martín J., Carrascal T., Walsh P., Reznikov L.L., Kim S.-H. (2000). IL-18 regulates IL-1beta -dependent hepatic melanoma metastasis via vascular cell adhesion molecule-1. Proc. Natl. Acad. Sci. USA.

[197] Guo B., Fu S., Zhang J., Liu B., Li Z. (2016). Targeting inflammasome/IL-1 pathways for cancer immunotherapy. Sci. Rep..

[198] Holen I., Lefley D.V., Francis S.E., Rennicks S., Bradbury S., Coleman R.E., Ottewell P. (2016). IL-1 drives breast cancer growth and bone metastasis in vivo. Oncotarget.

[199] Liu Q., Russell M.R., Shahriari K., Jernigan D., Lioni M.I., Garcia F.U., Fatatis A. (2013). Interleukin-1 Promotes Skeletal Colonization and Progression of Metastatic Prostate Cancer Cells with Neuroendocrine Features. Cancer Res..

[200] Liao T.-T., Yang M.-H. (2017). Revisiting epithelial-mesenchymal transition in cancer metastasis: The connection between epithelial plasticity and stemness. Mol. Oncol..

[201] Li R., Ong S.L., Tran L.M., Jing Z., Liu B., Park S.J., Huang Z.L., Walser T.C., Heinrich E.L., Lee G. (2020). Chronic IL-1β-induced inflammation regulates epithelial-to-mesenchymal transition memory phenotypes via epigenetic modifications in non-small cell lung cancer. Sci. Rep..

[202] Jiménez-Garduño A.M., Mendoza-Rodríguez M.G., Urrutia-Cabrera D., Domínguez-Robles M.C., Pérez-Yepez E.A., Ayala-Sumuano J.T., Meza I. (2017). IL-1β induced methylation of the estrogen receptor ERα gene correlates with EMT and chemoresistance in breast cancer cells. Biochem. Biophys. Res. Commun..

[203] Mendoza-Rodríguez M., Romero H.A., Fuentes-Pananá E.M., Ayala-Sumuano J.-T., Meza I. (2017). IL-1β induces up-regulation of BIRC3, a gene involved in chemoresistance to doxorubicin in breast cancer cells. Cancer Lett..

[204] Kaler P., Galea V., Augenlicht L., Klampfer L. (2010). Tumor Associated Macrophages Protect Colon Cancer Cells from TRAIL-Induced Apoptosis through IL-1β- Dependent Stabilization of Snail in Tumor Cells. PLoS ONE.

[205] Castaño Z., Juan B.P.S., Spiegel A., Pant A., de Cristo M.J., Laszewski T., Ubellacker J.M., Janssen S.R., Dongre A., Reinhardt F. (2018). IL-1β inflammatory response driven by primary breast cancer prevents metastasis-initiating cell colonization. Nature.

[206] Li Y., Wang L., Pappan L., Galliher-Beckley A., Shi J. (2012). IL-1β promotes stemness and invasiveness of colon cancer cells through Zeb1 activation. Mol. Cancer.

[207] Li H.-J., Reinhardt F., Herschman H.R., A Weinberg R. (2012). Cancer-stimulated mesenchymal stem cells create a carcinoma stem cell niche via prostaglandin E2 signaling. Cancer Discov..

[208] Yu A., Wang Y., Bian Y., Chen L., Guo J., Shen W., Chen D., Liu S., Sun X. (2018). IL-1β promotes the nuclear translocaiton of S100A4 protein in gastric cancer cells MGC803 and the cell’s stem-like properties through PI3K pathway. J. Cell. Biochem..

[209] Fei F., Qu J., Zhang S., Li Y., Zhang S. (2017). S100A4 in cancer progression and metastasis: A systematic review. Oncotarget.

[210] Watanabe T., Hashimoto T., Sugino T., Soeda S., Nishiyama H., Morimura Y., Yamada H., Goodison S., Fujimori K. (2012). Production of IL1-beta by ovarian cancer cells induces mesothelial cell beta1-integrin expression facilitating peritoneal dissemination. J. Ovarian Res..

[211] Hübner M., Effinger D., Wu T., Strauß G., Pogoda K., Kreth F.-W., Kreth S. (2020). The IL-1 Antagonist Anakinra Attenuates Glioblastoma Aggressiveness by Dampening Tumor-Associated Inflammation. Cancers.

[212] Lee H.E., Lee J.Y., Yang G., Kang H.C., Cho Y.-Y., Lee H.S., Lee J.Y. (2019). Inhibition of NLRP3 inflammasome in tumor microenvironment leads to suppression of metastatic potential of cancer cells. Sci. Rep..

[213] Storr S.J., Safuan S., Ahmad N., El-Refaee M., Jackson A.M., Martin S.G. (2017). Macrophage-derived interleukin-1beta promotes human breast cancer cell migration and lymphatic adhesion in vitro. Cancer Immunol. Immunother..

[214] Ershaid N., Sharon Y., Doron H., Raz Y., Shani O., Cohen N., Monteran L., Leider-Trejo L., Ben-Shmuel A., Yassin M. (2019). NLRP3 inflammasome in fibroblasts links tissue damage with inflammation in breast cancer progression and metastasis. Nat. Commun..

[215] Wei L.-Y., Lee J.-J., Yeh C.-Y., Yang C.-J., Kok S.-H., Ko J.-Y., Tsai F.-C., Chia J.-S. (2019). Reciprocal activation of cancer-associated fibroblasts and oral squamous carcinoma cells through CXCL1. Oral Oncol..

[216] Li S., Huang C., Hu G., Ma J., Chen Y., Zhang J., Huang Y., Zheng J., Xue W., Xu Y. (2020). Tumor-educated B cells promote renal cancer metastasis via inducing the IL-1β/HIF-2α/Notch1 signals. Cell Death Dis..

[217] Huang Q., Lan F.-H., Wang X., Yu Y., Ouyang X., Zheng F., Han J., Lin Y., Xie Y., Xie F. (2014). IL-1β-induced activation of p38 promotes metastasis in gastric adenocarcinoma via upregulation of AP-1/c-fos, MMP2 and MMP9. Mol. Cancer.

[218] Ma L., Lan F.-H., Zheng Z., Xie F., Wang L., Liu W., Han J., Zheng F., Xie Y., Huang Q. (2012). Epidermal growth factor (EGF) and interleukin (IL)-1β synergistically promote ERK1/2-mediated invasive breast ductal cancer cell migration and invasion. Mol. Cancer.

[219] Franco-Barraza J., Valdivia-Silva J., Zamudio-Meza H., Castillo A., Garcia-Zepeda E.A., Benitez-Bribiesca L., Meza I. (2010). Actin Cytoskeleton Participation in the Onset of IL-1β Induction of an Invasive Mesenchymal-like Phenotype in Epithelial MCF-7 Cells. Arch. Med Res..

[220] Guo R., Qin Y., Shi P., Xie J., Chou M., Chen Y. (2016). IL-1β promotes proliferation and migration of gallbladder cancer cells via Twist activation. Oncol. Lett..

[221] Pérez-Yépez E.A., Ayala-Sumuano J.-T., Lezama R., Meza I. (2014). A novel β-catenin signaling pathway activated by IL-1β leads to the onset of epithelial–mesenchymal transition in breast cancer cells. Cancer Lett..

[222] Westbom C., Thompson J.K., Leggett A., MacPherson M., Beuschel S., Pass H.I., Vacek P., Shukla A. (2015). Inflammasome Modulation by Chemotherapeutics in Malignant Mesothelioma. PLoS ONE.

[223] Arlt A., Vorndamm J., Müerköster S., Yu H., Schmidt W.E., Fölsch U.R., Schäfer H. (2002). Autocrine production of interleukin 1beta confers constitutive nuclear factor kappaB activity and chemoresistance in pancreatic carcinoma cell lines. Cancer Res..

[224] Müerköster S.S., Werbing V., Sipos B., A Debus M., Witt M., Großmann M., Leisner D., Kötteritzsch J., Kappes H., Klöppel G. (2006). Drug-induced expression of the cellular adhesion molecule L1CAM confers anti-apoptotic protection and chemoresistance in pancreatic ductal adenocarcinoma cells. Oncogene.

[225] Müerköster S., Wegehenkel K., Arlt A., Witt M., Sipos B., Kruse M.-L., Sebens T., Klöppel G., Kalthoff H., Fölsch U.R. (2004). Tumor Stroma Interactions Induce Chemoresistance in Pancreatic Ductal Carcinoma Cells Involving Increased Secretion and Paracrine Effects of Nitric Oxide and Interleukin-1. Cancer Res..

[226] Arlt A., Vorndamm J., Witt M., Grohmann F. (2004). Autokrine IL-1β-Sekretion führt zu erhöhter NF-κB-Aktivität und zu Chemoresistenz in Pankreaskarzinomzellen in vivo. Med. Klin..

[227] Jin H., Ko Y.S., Kim H.J. (2018). P2Y2R-mediated inflammasome activation is involved in tumor progression in breast cancer cells and in radiotherapy-resistant breast cancer. Int. J. Oncol..

[228] Ghiringhelli F., Apetoh L., Tesniere A., Aymeric L., Ma Y., Ortiz C., Vermaelen K., Panaretakis T., Mignot G., Ullrich E. (2009). Activation of the NLRP3 inflammasome in dendritic cells induces IL-1β–dependent adaptive immunity against tumors. Nat. Med..

[229] Mattarollo S., Loi S., Duret H., Ma Y., Zitvogel L., Smyth M.J. (2011). Pivotal Role of Innate and Adaptive Immunity in Anthracycline Chemotherapy of Established Tumors. Cancer Res..

[230] Bruchard M., Mignot G., Derangere V., Chalmin F., Chevriaux A., Vegran F., Boireau W., Simon B., Ryffel B., Connat J.L. (2012). Chemotherapy-triggered cathepsin B release in myeloid-derived suppressor cells activates the Nlrp3 inflammasome and promotes tumor growth. Nat. Med..

[231] Isambert N., Hervieu A., Rébé C., Hennequin A., Borg C., Zanetta S., Chevriaux A., Richard C., Derangère V., Limagne E. (2018). Fluorouracil and bevacizumab plus anakinra for patients with metastatic colorectal cancer refractory to standard therapies (IRAFU): A single-arm phase 2 study. OncoImmunology.

[232] Martine P., Chevriaux A., Derangère V., Apetoh L., Garrido C., Ghiringhelli F., Rébé C. (2019). HSP70 is a negative regulator of NLRP3 inflammasome activation. Cell Death Dis..

[233] Martine P. (2019). Heat Shock Proteins and Inflammasomes. Int. J. Mol. Sci..

[234] Pilot T., Fratti A., Thinselin C., Perrichet A., Demontoux L., Limagne E., Derangère V., Ilie A., Ndiaye M., Jacquin E. (2020). Heat shock and HSP70 regulate 5-FU-mediated caspase-1 activation in myeloid-derived suppressor cells and tumor growth in mice. J. Immunother. Cancer.

[235] Son S., Shim D.-W., Hwang I., Park J.-H., Yu J.-W. (2019). Chemotherapeutic Agent Paclitaxel Mediates Priming of NLRP3 Inflammasome Activation. Front. Immunol..

[236] Zeng Q.-Z., Yang F., Li C.-G., Xu L.-H., He X.-H., Mai F.-Y., Zeng C.-Y., Zhang C.-C., Zha Q.-B., Ouyang D.-Y. (2019). Paclitaxel Enhances the Innate Immunity by Promoting NLRP3 Inflammasome Activation in Macrophages. Front. Immunol..

[237] Voloshin T., Alishekevitz D., Kaneti L., Miller V., Isakov E., Kaplanov I., Voronov E., Fremder E., Benhar M., Machluf M. (2015). Blocking IL1 Pathway Following Paclitaxel Chemotherapy Slightly Inhibits Primary Tumor Growth but Promotes Spontaneous Metastasis. Mol. Cancer Ther..

[238] Waugh J., Perry C.M. (2005). Anakinra. BioDrugs.

[239] Lust J.A., Lacy M.Q., Zeldenrust S.R., Dispenzieri A., Gertz M.A., Witzig T.E., Kumar S., Hayman S.R., Russell S.J., Buadi F.K. (2009). Induction of a Chronic Disease State in Patients With Smoldering or Indolent Multiple Myeloma by Targeting Interleukin 1β-Induced Interleukin 6 Production and the Myeloma Proliferative Component. Mayo Clin. Proc..

[240] Dubois E.A., Rissmann R., Cohen A.F. (2011). Rilonacept and canakinumab. Br. J. Clin. Pharmacol..

[241] Ridker P.M., MacFadyen J.G., Everett B.M., Ridker P.M., Lorenzatti A., Krum H., Varigos J., Siostrzonek P., Sinnaeve P., Fonseca F. (2017). Effect of interleukin-1β inhibition with canakinumab on incident lung cancer in patients with atherosclerosis: Exploratory results from a randomised, double-blind, placebo-controlled trial. Lancet.

[242] van Cutsem E., Shitara K., Deng W., Vaury A., Tseng L., Wang X., Millholland J., Shilkrut M., Mookerjee B., Jonasch E. (2019). Gevokizumab, an interleukin-1β (IL-1β) monoclonal antibody (mAb), in metastatic colorectal cancer (mCRC), metastatic gastroesophageal cancer (mGEC) and metastatic renal cell carcinoma (mRCC): “First-in-cancer” phase Ib study. Ann. Oncol..

[243] Yang Y., Wang H., Kouadir M., Song H., Shi F. (2019). Recent advances in the mechanisms of NLRP3 inflammasome activation and its inhibitors. Cell Death Dis..

[244] Perera P., Fernando R., Shinde T., Gundamaraju R., Southam B., Sohal S.S., Robertson A.A.B., Schroder K., Kunde D., Eri R. (2018). MCC950, a specific small molecule inhibitor of NLRP3 inflammasome attenuates colonic inflammation in spontaneous colitis mice. Sci. Rep..

[245] Scarpellini M., Lurati A., Vignati G., Marrazza M.G., Telese F., Re K., Bellistri A. (2008). Biomarkers, type II collagen, glucosamine, and chondroitin sulfate in osteoarthritis follow-up: The “Magenta osteoarthritis study. ” J. Orthop. Traumatol..

[246] Wannamaker W., Davies R., Namchuk M., Pollard J., Ford P., Ku G., Decker C., Charifson P., Weber P., Germann U.A. (2007). (S)-1-((S)-2-{[1-(4-Amino-3-chloro-phenyl)-methanoyl]-amino}-3,3-dimethyl-butanoyl)-pyrrolidine-2-carboxylic acid ((2R,3S)-2-ethoxy-5-oxo-tetrahydro-furan-3-yl)-amide (VX-765), an Orally Available Selective Interleukin (IL)-Converting Enzyme/Caspase-1 Inhibitor, Exhibits Potent Anti-Inflammatory Activities by Inhibiting the Release of IL-1β and IL-18. J. Pharmacol. Exp. Ther..

[247] Schroder K., Sagulenko V., Zamoshnikova A., Richards A.A., Cridland J.A., Irvine K., Stacey K.J., Sweet M.J. (2012). Acute lipopolysaccharide priming boosts inflammasome activation independently of inflammasome sensor induction. Immunobiology.

[248] Helal K.F., Badr M.S., Rafeek M.E.-S., Elnagar W.M., Lashin M.E.-B. (2020). Can glyburide be advocated over subcutaneous insulin for perinatal outcomes of women with gestational diabetes? A systematic review and meta-analysis. Arch. Gynecol. Obstet..

[249] Adinolfi E., Raffaghello L., Giuliani A.L., Cavazzini L., Capece M., Chiozzi P., Bianchi G., Kroemer G., Pistoia V., di Virgilio F. (2012). Expression of P2X7 Receptor Increases In Vivo Tumor Growth. Cancer Res..

